# The Impact of eHealth on the Quality and Safety of Health Care: A Systematic Overview

**DOI:** 10.1371/journal.pmed.1000387

**Published:** 2011-01-18

**Authors:** Ashly D. Black, Josip Car, Claudia Pagliari, Chantelle Anandan, Kathrin Cresswell, Tomislav Bokun, Brian McKinstry, Rob Procter, Azeem Majeed, Aziz Sheikh

**Affiliations:** 1 eHealth Unit, Department of Primary Care and Public Health, Imperial College London, London, United Kingdom; 2 eHealth Research Group, Centre for Population Health Sciences, The University of Edinburgh, Edinburgh, United Kingdom; 3 National Centre for e-Social Science, University of Manchester, Manchester, United Kingdom; 4 Department of Primary Care and Public Health, Imperial College London, London, United Kingdom; University of South Florida, United States of America

## Abstract

**Background:**

There is considerable international interest in exploiting the potential of digital solutions to enhance the quality and safety of health care. Implementations of transformative eHealth technologies are underway globally, often at very considerable cost. In order to assess the impact of eHealth solutions on the quality and safety of health care, and to inform policy decisions on eHealth deployments, we undertook a systematic review of systematic reviews assessing the effectiveness and consequences of various eHealth technologies on the quality and safety of care.

**Methods and Findings:**

We developed novel search strategies, conceptual maps of health care quality, safety, and eHealth interventions, and then systematically identified, scrutinised, and synthesised the systematic review literature. Major biomedical databases were searched to identify systematic reviews published between 1997 and 2010. Related theoretical, methodological, and technical material was also reviewed. We identified 53 systematic reviews that focused on assessing the impact of eHealth interventions on the quality and/or safety of health care and 55 supplementary systematic reviews providing relevant supportive information. This systematic review literature was found to be generally of substandard quality with regards to methodology, reporting, and utility. We thematically categorised eHealth technologies into three main areas: (1) storing, managing, and transmission of data; (2) clinical decision support; and (3) facilitating care from a distance. We found that despite support from policymakers, there was relatively little empirical evidence to substantiate many of the claims made in relation to these technologies. Whether the success of those relatively few solutions identified to improve quality and safety would continue if these were deployed beyond the contexts in which they were originally developed, has yet to be established. Importantly, best practice guidelines in effective development and deployment strategies are lacking.

**Conclusions:**

There is a large gap between the postulated and empirically demonstrated benefits of eHealth technologies. In addition, there is a lack of robust research on the risks of implementing these technologies and their cost-effectiveness has yet to be demonstrated, despite being frequently promoted by policymakers and “techno-enthusiasts” as if this was a given. In the light of the paucity of evidence in relation to improvements in patient outcomes, as well as the lack of evidence on their cost-effectiveness, it is vital that future eHealth technologies are evaluated against a comprehensive set of measures, ideally throughout all stages of the technology's life cycle. Such evaluation should be characterised by careful attention to socio-technical factors to maximise the likelihood of successful implementation and adoption.

*Please see later in the article for the Editors' Summary*

## Abstract 2

Aziz Sheikh and colleagues report the findings of their systematic overview that assessed the impact of eHealth solutions on the quality and safety of health care.

## Editors' Summary

**Background:**

There is considerable international interest in exploiting the potential of digital health care solutions, often referred to as eHealth—the use of information and communication technologies—to enhance the quality and safety of health care. Often accompanied by large costs, any large-scale expenditure on eHealth—such as electronic health records, picture archiving and communication systems, ePrescribing, associated computerized provider order entry systems, and computerized decision support systems—has tended to be justified on the grounds that these are efficient and cost-effective means for improving health care. In 2005, the World Health Assembly passed an eHealth resolution (WHA 58.28) that acknowledged, “eHealth is the cost-effective and secure use of information and communications technologies in support of health and health-related fields, including health-care services, health surveillance, health literature, and health education, knowledge and research,” and urged member states to develop and implement eHealth technologies. Since then, implementing eHealth technologies has become a main priority for many countries. For example, England has invested at least £12.8 billion in a National Programme for Information Technology for the National Health Service, and the Obama administration in the United States has committed to a US$38 billion eHealth investment in health care.

**Why Was This Study Done?:**

Despite the wide endorsement of and support for eHealth, the scientific basis of its benefits—which are repeatedly made and often uncritically accepted—remains to be firmly established. A robust evidence-based perspective on the advantages on eHealth could help to suggest priority areas that have the greatest potential for benefit to patients and also to inform international eHealth deliberations on costs. Therefore, in order to better inform the international community, the authors systematically reviewed the published systematic review literature on eHealth technologies and evaluated the impact of these technologies on the quality and safety of health care delivery.

**What Did the Researchers Do and Find?:**

The researchers divided eHealth technologies into three main categories: (1) storing, managing, and transmission of data; (2) clinical decision support; and (3) facilitating care from a distance. Then, implementing methods based on those developed by the Cochrane Collaboration and the NHS Service Delivery and Organisation Programme, the researchers used detailed search strategies and maps of health care quality, safety, and eHealth interventions to identify relevant systematic reviews (and related theoretical, methodological, and technical material) published between 1997 and 2010. Using these techniques, the researchers retrieved a total of 46,349 references from which they identified 108 reviews. The 53 reviews that the researchers finally selected (and critically reviewed) provided the main evidence base for assessing the impact of eHealth technologies in the three categories selected.

In their systematic review of systematic reviews, the researchers included electronic health records and picture archiving communications systems in their evaluation of category 1, computerized provider (or physician) order entry and e-prescribing in category 2, and all clinical information systems that, when used in the context of eHealth technologies, integrate clinical and demographic patient information to support clinician decision making in category 3.

The researchers found that many of the clinical claims made about the most commonly used eHealth technologies were not substantiated by empirical evidence. The evidence base in support of eHealth technologies was weak and inconsistent and importantly, there was insubstantial evidence to support the cost-effectiveness of these technologies. For example, the researchers only found limited evidence that some of the many presumed benefits could be realized; importantly, they also found some evidence that introducing these new technologies may on occasions also generate new risks such as prescribers becoming over-reliant on clinical decision support for e-prescribing, or overestimate its functionality, resulting in decreased practitioner performance.

**What Do These Findings Mean?:**

The researchers found that despite the wide support for eHealth technologies and the frequently made claims by policy makers when constructing business cases to raise funds for large-scale eHealth projects, there is as yet relatively little empirical evidence to substantiate many of the claims made about eHealth technologies. In addition, even for the eHealth technology tools that have proven to be successful, there is little evidence to show that such tools would continue to be successful beyond the contexts in which they were originally developed. Therefore, in light of the lack of evidence in relation to improvements in patient outcomes, as well as the lack of evidence on their cost-effectiveness, the authors say that future eHealth technologies should be evaluated against a comprehensive set of measures, ideally throughout all stages of the technology's life cycle, and include socio-technical factors to maximize the likelihood of successful implementation and adoption in a given context. Furthermore, it is equally important that eHealth projects that have already been commissioned are subject to rigorous, multidisciplinary, and independent evaluation.

**Additional Information:**

Please access these websites via the online version of this summary at http://dx.doi.org/10.1371/journal.pmed.1000387.

- The authors' broader study is: Car J, Black A, Anandan C, Cresswell K, Pagliari C, McKinstry B, et al. (2008) The Impact of eHealth on the Quality and Safety of Healthcare. Available at: http://www.haps.bham.ac.uk/publichealth/cfhep/001.shtml
- More information is available on the World Health Assembly eHealth resolution
- The World Health Organization provides information at the Global Observatory on eHealth, as well as a global insight into eHealth developments
- The European Commission provides Information on eHealth in Europe and some examples of good eHealth practice
- More information is provided on NHS Connecting for Health

## Introduction

Implementations of potentially transformative eHealth technologies are currently underway internationally, often with significant impact on national expenditure. England has, for example, invested at least £12.8 billion in a National Programme for Information Technology (NPfIT) for the National Health Service, and the Obama administration in the United States (US) has similarly committed to a US$38 billion eHealth investment in health care [1]. Such large-scale expenditure has been justified on the grounds that electronic health records (EHRs), picture archiving and communication systems (PACS), electronic prescribing (ePrescribing) and associated computerised provider (or physician) order entry systems (CPOE), and computerised decision support systems (CDSSs) will help address the problems of variable quality and safety in modern health care. However, the scientific basis of such claims—which are repeatedly made and seemingly uncritically accepted—remains to be established [2]–[7].

Moving this agenda forward thus requires a scientifically informed perspective. However, there remains a disparity between the evidence-based principles that underpin health care generally and the political, pragmatic, and commercial drivers of decision making in the commissioning of eHealth tools and services. Obtaining an evidence-informed perspective on the current situation may serve to ground unrealistic expectations that might hinder longer-term progress within the field, help to suggest priorities by identifying areas with greatest potential for benefit, and also inform ongoing deliberations on eHealth implementations that are being considered internationally.

To inform these global deliberations, we systematically reviewed the preexisting systematic review literature on eHealth technologies and their impact on the quality and safety of health care delivery. We synthesised and contextualised our findings with the broader theoretical and methodological literature with a view to producing a comprehensive and accessible overview of the field. We present here a synopsis and updated version of a much larger recently published report covering the period 1997–2010 [8].

## Methods

### Overview of Methods

Systematic reviews of reviews have been particularly advocated to inform policy, clinical, and research deliberations by providing an evidence-based summary of inter-related technologies [9]. Our approach involved drawing on established systematic review methodology (i.e., those developed by The Cochrane Collaboration) to ensure rigour by minimising the risk of bias [10]; we also drew on more novel methods of evidence synthesis (i.e., those developed by the UK National Health Service [NHS] Service Delivery and Organisation Programme) with the aim of producing an overview that we hoped would prove useful to decision makers [11]. We present here a summary of the methods used.

### Developmental Work

Inherent difficulties associated with systematic reviews of health care organisation and delivery intervention include the considerable effort required at the outset to facilitate their conduct [9]. Accordingly, we began with an in-depth exploration of the fields of health care quality and safety, as well as eHealth functionalities used in health care delivery. This exploration entailed conceptually mapping the fields to understand various processes involved as well as how these relate to each other.

For quality and safety considerations, we identified existing taxonomies and frameworks to facilitate this conceptual mapping exercise, which helped to delineate the scope of our work. For the field of eHealth, we drew from existing team members' conceptual and empirical work to aid our construction of a conceptual map for eHealth technologies [12],[13]. This exercise allowed us to categorise interventions with regards to over-arching similarities. We characterised eHealth technologies as having three main overlapping functions: (1) to enable the storage, retrieval, and transmission of data; (2) to support clinical decision making; and (3) to facilitate remote care. Given the strategic focus of the English National Programme for Information Technology (NPfIT) (and other similar large-scale programmes) on electronic record and professional decision support systems [1], the first two functions were prioritised in this initial phase of our work. The current reported work thus concerns the related areas of EHRs, PACS, CPOEs, ePrescribing, and computerised systems for supporting clinical decision making. Remote care and consumer health informatics are the subjects of a subsequent 3-y research enquiry, which is currently in progress.

### Search Strategy

We drew on established Cochrane-based systematic review principles to search for relevant systematic reviews. An inclusive string of MeSH and free terms (Text S1) was developed to query PubMed/MEDLINE, EMBASE, and the Cochrane Library contents for secondary research reports published from 1997 up to 2007 with no restrictions placed on language. The bibliographies of reports identified as potentially relevant were reviewed as was a catalogue of secondary research amassed through various contributions by team members. Additional searches of key health informatics resources, namely the conference proceedings and publication databases of the American Medical Informatics Association and the Agency of Healthcare Research and Quality, were also undertaken. Finally, the Internet was searched using the Google and Google Scholar search engines. Searches were periodically updated to ensure that the most recent publications were included with the last update occurring at the end of April 2010.

### Selection and Critical Appraisal of Systematic Reviews

On the basis of the areas identified for prioritisation, we developed a detailed list of interventions that were to be included/excluded (Text S2). End users of applicable interventions were limited to health care professionals; any findings relating to patient-focused interventions were therefore excluded. Of interest were systematic reviews that focused on the assessment of patient, practitioner, or organisational outcomes. We detailed the following methodological criteria for the identification of systematic reviews: (1) reference to the study as being a systematic review by the authors within the title, abstract, or text; and/or (2) evidence from the description of the methods that systematic review principles had been utilised in searching and appraising the evidence.

All systematic reviews having been identified as potentially suitable were assessed for inclusion by two independent reviewers, with arbitration by a third reviewer if necessary. Data from systematic reviews meeting the above criteria, henceforth referred to as “reviews,” were independently critically reviewed by two reviewers, and relevant data were abstracted. Systematic reviews not primarily concerned with assessing impact on patients, professionals, or the organisation, but nonetheless intervention focused, were drawn on to provide additional contextual information. These supplementary systematic reviews (henceforth referred to as “supplementary reviews”) were not subjected to formal critical appraisal.

Critical appraisal was undertaken using an adapted version of the Critical Appraisal Skills Programme (CASP) tool for systematic reviews [14]. These modifications were informed by the growing literature regarding both the methodological and reporting issues with primary research in health informatics (Table S1). The details of this process and the tool's associated properties will be the subject of a separate publication in due course.

### Data Synthesis

A standard approach was taken for each of the eHealth technologies of interest. Definitions were first clarified and then the individual use and broader scope for deployment conceptualised. Juxtaposing this with the aforementioned conceptual maps of the fields of eHealth, quality and safety provided a literature-based framework for delineating the principal theorised benefits and risks associated with each intervention. We used this framework to guide synthesis of the empirically demonstrated benefits and risks of implementing eHealth technologies.

The body of literature identified was too diverse to allow quantitative synthesis of empirical evidence and we therefore undertook a narrative synthesis. This synthesis involved initially describing the technologies and outcomes studies using the above-described framework for each of the included reviews, which was followed by developing a summary of our assessment of and the key findings from each review (Table S2). We then employed a modified version of the World Health Organization's Health Evidence Network system for appraising public health evidence, which classifies evidence into three main categories, i.e., strong, moderate or weak; this assessment being based on a combination of the overall consistency, quality, and volume of evidence uncovered. These review-derived data were then thematically synthesised in relation to each of the technologies under consideration, drawing on key findings from the additional reviews, as appropriate [8].

## Results

**Figure 1:**
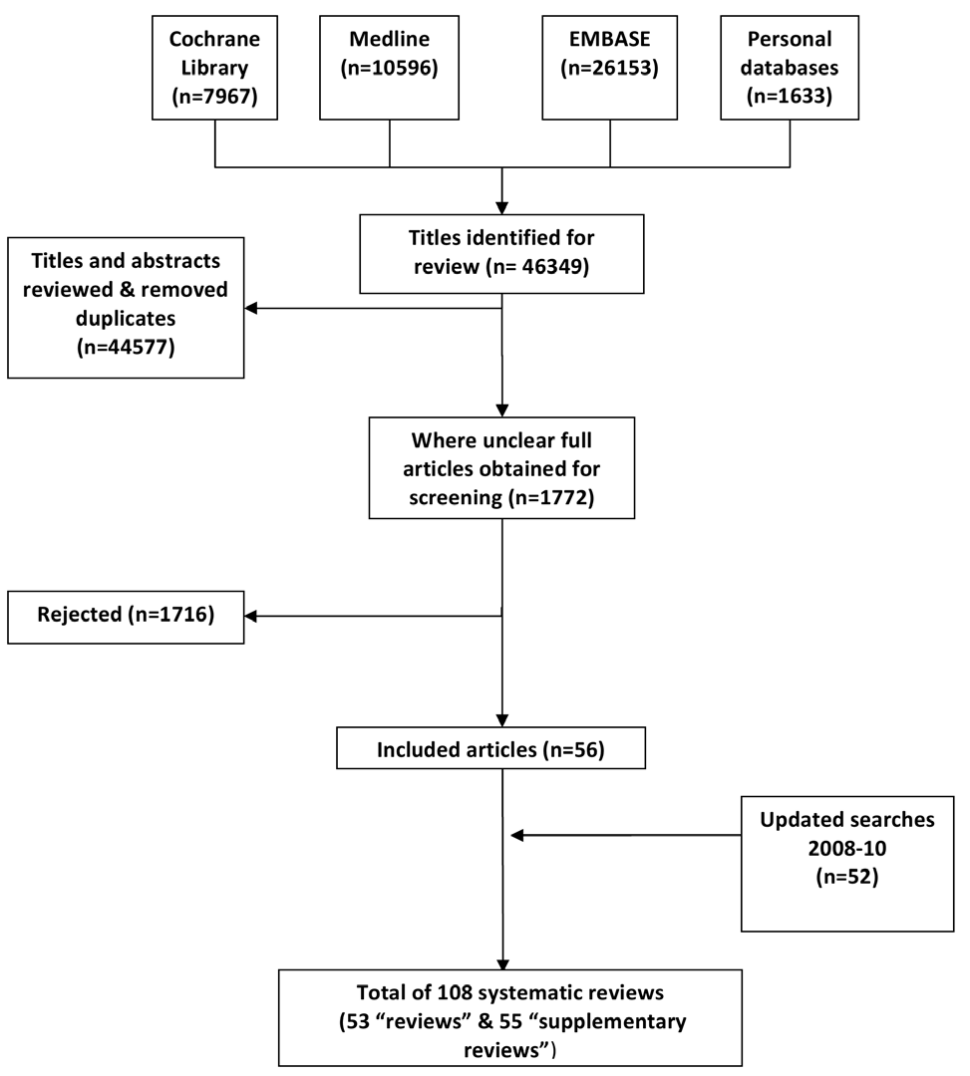
PRISMA flow diagram.

Our searches retrieved a total of 46,349 references from which we selected a total of 108 reviews for inclusion (Figure 1). Our final selection of 53 reviews provided the main empirical evidence base in relation to assessing the impact of the selected eHealth technologies (see Table 1 for our critical appraisal of these studies) [15]–[67], full details of which can be found in Table S2. An additional 55 supplementary reviews provided context to the findings [68]–[122], aiding in their interpretation [123]. In the case of systematic review updates, only the most recent review in a series of updates was selected. In the case of full and summary publications, we drew on the more substantive reports. Three related reviews – an update, a fuller report, and its more concise counterpart – were an exception due to the complementary nature of the reports rather than these being duplicative [22],[55],[56].

**Table 1:** Critical appraisal of “reviews” (see legend for description of quality assessment criteria).

Lead Author and Year	Q1	Q2	Q3	Q4	Q5	Q6	Q7	Q8	Q9	Q10	Q11	Q12	Q13	Q14	Q15	Total^a^
Ammenwerth 2008	2	2	2	1	2	2	2	1	2	2	1	2	1	1	1	24
Anderson 1997	2	2	2	1	2	1	1	2	1	2	1	2	1	1	2	23
Balas 2004	1	1	2	1	2	1	2	1	2	2	1	2	1	2	1	22
Bennett 2003	1	1	1	2	2	1	2	1	1	1	1	1	1	1	1	18
Bryan 2008	1	2	1	2	2	2	1	1	1	1	1	1	1	1	1	19
Charvet-Protat 1998	1	2	1	0	0	0	1	1	0	1	1	1	1	1	1	12
Chatellier 1998	2	2	2	0	1	1	2	2	2	2	1	1	1	1	1	21
Chaudhry 2006	1	1	2	2	0	1	1	1	2	1	0	2	0	2	2	18
Clamp 2005	1	1	2	0	1	1	1	1	2	1	1	1	1	2	1	17
Delpierre 2004	1	1	0	1	0	2	1	0	2	1	0	1	1	1	1	13
Dexheimer 2008	2	1	1	1	2	1	2	1	2	2	1	2	1	0	0	19
Durieux 2008	2	2	1	2	2	1	2	1	2	2	2	1	1	1	1	23
Eslami 2007	2	2	1	1	0	1	2	2	1	1	0	1	1	2	1	18
Eslami 2008	2	2	1	1	0	1	2	2	1	1	0	1	1	2	1	18
Eslami 2009	2	2	1	0	1	2	2	0	1	0	0	2	1	1	1	16
Fitzmaurice 1998	2	1	0	2	2	0	1	1	1	1	0	1	0	0	0	12
Garg 2005	1	1	2	2	2	1	1	1	2	2	1	1	1	1	1	20
Georgiou 2007	2	2	1	2	1	1	1	2	2	1	0	2	1	2	1	21
Hayward 2009	2	0	0	1	0	1	2	1	2	1	0	2	1	1	1	15
Hender 2000	1	1	2	1	2	1	1	1	1	0	0	0	0	0	0	11
Heselmans 2009	2	2	2	2	2	1	2	1	2	1	1	1	1	1	1	22
Hider 2002	1	1	2	2	1	2	1	1	2	2	1	2	1	1	1	21
Irani 2009	2	1	1	1	1	2	2	1	1	1	1	1	1	1	1	18
Jamal 2009	1	1	2	2	2	1	1	1	2	1	0	1	1	0	0	16
Jerant 2000	1	2	0	0	2	1	1	1	2	1	0	2	1	1	1	16
Kaushal 2003	2	2	2	1	1	1	1	2	0	2	1	1	0	0	0	16
Mador 2009	2	2	1	2	2	2	2	2	1	0	0	1	1	1	1	20
Mitchell 2001	1	1	1	2	2	1	1	1	0	1	1	1	1	2	1	17
Montgomery 1998	2	2	2	1	1	1	1	1	1	1	0	0	1	0	0	14
Niazkhani 2009	2	2	2	1	0	1	1	2	2	1	1	2	1	2	2	22
Oren 2003	1	2	1	0	0	1	1	1	0	1	0	1	1	2	2	14
Pearson 2009	2	2	2	1	2	2	2	2	2	1	1	1	1	1	1	23
Poissant 2005	2	1	2	2	2	1	2	1	2	2	2	2	1	2	1	25
Randell 2007	1	1	1	2	2	1	2	1	1	1	0	1	1	1	1	17
Reckmann 2009	2	2	1	2	0	2	1	1	1	1	0	2	1	2	2	20
Rothschild 2004	1	1	1	1	0	1	2	1	2	1	0	2	1	1	1	16
Schedlbauer 2009	1	2	2	1	2	2	2	1	2	1	1	2	1	1	1	22
Shachak 2009	2	1	1	0	0	0	2	1	1	1	1	2	1	1	1	15
Shamliyan 2008	1	2	1	1	1	2	0	1	1	1	1	1	0	0	0	13
Shebl 2007	1	1	2	1	1	1	1	1	2	1	1	1	1	1	1	17
Shekelle 2006	1	1	2	1	1	2	1	1	2	1	1	2	1	2	1	20
Shekelle 2009	1	1	2	1	0	2	1	1	2	1	1	2	2	2	2	21
Shiffman 1999	1	1	2	0	0	2	1	1	1	1	0	1	1	1	1	14
Shojania 2009	1	2	2	1	2	2	2	1	2	2	2	1	1	1	0	22
Sintchenko 2007	1	1	1	0	1	1	2	1	2	2	1	1	1	1	1	17
Smith 2007	1	2	0	1	1	2	1	1	1	1	0	2	1	2	1	17
Tan 2005	2	2	1	2	2	2	2	2	2	1	0	1	0	0	0	19
Thompson 2009	2	1	1	1	0	0	1	2	0	1	0	0	0	0	0	9
Uslu 2008	2	1	0	0	2	1	2	2	1	1	1	1	0	1	1	16
van Rosse 2009	2	2	1	1	2	2	2	0	1	2	2	2	1	2	2	24
Wolfstadt 2008	1	1	0	0	2	2	1	1	1	1	1	1	1	0	0	13
Wong 2010	2	2	2	1	1	1	1	2	2	2	2	1	1	1	1	22
Yourman 2008	1	1	1	1	1	1	2	1	2	2	1	1	1	0	0	16
**Average scores**	**1.49**	**1.47**	**1.3**	**1.09**	**1.19**	**1.28**	**1.45**	**1.19**	**1.43**	**1.21**	**0.7**	**1.32**	**0.87**	**1.08**	**0.91**	**18**

Maximum total score of 30, each question *(or Q)* having a maximum of 2 points, refer to Table S1 for the critical appraisal form for additional details.

### Data Storage, Management, and Retrieval Systems

#### Electronic health records

The EHR is a complex construct encompassing digitised health care records and the information systems into which these are embedded [8]. Whilst there are a number of operational definitions, the US' Institute of Standards and Technology defines an EHR as “a longitudinal collection of patient-centric health care information available across providers, care settings, and time. It is a central component of an integrated health information system” [124]. EHRs can be used for the digital input, storage, display, retrieval, printing, and sharing of information contained in a patient's health record [8]. We found that these systems vary on multiple dimensions, including levels of sophistication, detail, data source, timeframe (single service encounter to complete health record), and extent of integration (across intra- and interservice boundaries). In addition to patient histories and details of recent care, these records may also incorporate digital images and scanned documents. More detailed EHRs further often include nonclinical data relevant to health care administration and/or planning such as, for example, bed management and commissioning data. EHRs can therefore be used by a variety of end users such as clinicians, administrators, and patients themselves. EHRs can also have varying degrees of added clinical functionality including the ability to interface with a digital PACS, enter orders electronically (i.e., CPOE), prescribing (ePrescribing), and access to CDSSs.

The theorised benefits and risks associated with EHRs are largely related to data storage and management functionality. These functions include increased accessibility, legibility, “searchability,” manipulation, transportation, sharing, and preservation of electronic data. Consequently, improved organisational efficiency and secondary uses of data are typically amongst the most commonly expected benefits. However, digitising health records can also introduce new risks. Paper persistence can result in threats to patient safety, unsecured networks can lead to illegitimate access, and increased time needed to document and retrieve patient data can result in organisational inefficiency. Moreover, the dynamic of the patient-provider interaction could become less personal with the intrusion by the computer as a “third person” in the consultation. If anticipated benefits are not realised, this may therefore mean that ultimately the EHR may be rendered cost-ineffective.

Although a number of reviews purporting to assess the impact of EHRs were found, many of these in fact investigated auxiliary systems such as CDSS, CPOE, and ePrescribing. As a result, most of the impacts assessed were more relevant to these other systems. We found only anecdotal evidence of the fundamental expected benefits and risks relating to the organisational efficiency resulting from the storage and management facilities within the EHR and thus the potential for secondary uses (Table 2). We did find, however, a small amount of secondary research relating to time efficiency for some health care professionals and administrators and data quality (in particular legibility, completeness, and comprehensiveness), which demonstrated weak evidence of benefit for both. Risks largely went ignored apart from anecdotal evidence of time-costs associated with recording of data due to both end-user skill and the inflexibility of structured data, increased costs of EHRs, and a decrease in patient-centeredness within the consultation (Table 3).

**Table 2:** Evidence of benefits associated with EHRs.

Benefits							
Review ID	Data Security	Legibility	Accessibility	Completeness	Comprehensiveness	Efficiency	Secondary Uses
Clamp 2005	N/A	+	+	+	N/A	+/-	N/A
Irani 2009	N/A	N/A	N/A	N/A	N/A	N/A	N/A
Jamal 2009	N/A	N/A	N/A	+/++	N/A	+	N/A
Mador 2009	N/A	N/A	++	N/A	N/A	+/-	+/-
Mitchell 2001	+/-	+	N/A	+	+	+/+ +	N/A
Poissant 2005	N/A	N/A	N/A	N/A	N/A	+/+ +	N/A
Shachak 2009	N/A	N/A	N/A	+	+/++	N/A	N/A
Shekelle 2006	N/A	+	N/A	+	+	+/+ +	N/A
Shekelle 2009	N/A	N/A	N/A	N/A	N/A	+/-	+
Thompson 2009	N/A	N/A	N/A	N/A	N/A	+	N/A
Uslu 2008	N/A	+	+	N/A	N/A	++	N/A

Evidence of benefits: N/A, not assessed; +/-, none; +, weak; +/++, weak to moderate; ++, moderate.

**Table 3 pmed-1000387-t003:** Evidence of risks associated with EHRs.

Risks					
Review ID	Paper Persistence	Patient Disengagement	Insecure Data	Increased Time	Increased Costs
Clamp 2005	N/A	-	N/A	-	-
Irani 2009	N/A	+/-	N/A	N/A	N/A
Jamal 2009	N/A	N/A	N/A	+/-	+/-
Mador 2009	N/A	N/A	N/A	-	N/A
Mitchell 2001	N/A	-	-	-	-
Poissant 2005	N/A	N/A	N/A	- -	N/A
Shachak 2009	-	-/- -	N/A	N/A	N/A
Shekelle 2006	N/A	N/A	N/A	-	-
Shekelle 2009	N/A	N/A	N/A	-	-
Thompson 2009	N/A	N/A	N/A	+/-	N/A
Uslu 2008	N/A	N/A	N/A	N/A	+/-

Evidence of risks: N/A, not assessed; +/-, none; -, weak; -/- -, weak to moderate; --, moderate.

#### Picture archiving and communication systems

PACS are clinical information systems used for the acquisition, archival, and post-processing distribution of digital images. An image must either be directly acquired using digital radiography or be digitised from a paper-based format. It can be stored using an electronic, magnetic, or optical storage device. PACS can be integrated or interface with EHRs and CDSSs, or be stand-alone systems.

Much like the digitisation of health records, certain benefits – i.e., accessibility, image (rather than data) quality, searchability, transportation, sharing, and preservation – can be expected from the digitisation of medical images, which were previously film based. Again, certain improvements to organisational efficiency should in theory follow on from this digitisation, including time-savings, continuity of care, and ability to remotely view images. Conversely, digitising medical images can lead to decreased organisational efficiency if increased time is needed for retrieval owing to the difficulties associated with navigating a new or cumbersome system or in the event of system downtime. If the potential benefits of a PACS implementation are not realised, high expenditure might render the application cost-inefficient.

Although only three reviews on PACS were located, in contrast to the reviews on EHRs the impacts assessed in reviews of PACS were more congruent with the theoretically derived benefits (Table 4). This assessment involved a focus on improved organisational efficiency through time savings resulting from increased productivity of radiology services, reduced transit time, and improved access to new, recently stored, and archived images, as well as reducing physical space requirements for images; there was also an interest in the assessments of costs relating to purchasing and processing film. Worth noting however was the transient negative impact of implementation as well as issues with access due to system “loss” and downtime; access was sometimes impeded by the new workflows, which could result in a decrease in opportunistic interactions between clinicians and radiologists (Table 5). Overall, despite some promising findings, the weak evidence for the beneficial impact of digitising medical images is largely due to a low volume of research and somewhat inconsistent findings across studies. For example, the overall cost-effectiveness of systems could not be determined, as the findings from economic analyses were often contradictory and of poor quality.

**Table 4 pmed-1000387-t004:** Evidence of benefits associated with PACS.

Benefits						
Review ID	Data Integrity	Image Resolution	Image Access	Cost Savings	Time Savings	Diagnostic Accuracy
Anderson 1997	+	+/-	+	+/-	+	+/-
Charvet-Protat 1998	+	N/A	+	+	+	N/A
Clamp 2005	+	N/A	+	+/-	+	+

Evidence of benefits: N/A, not assessed; +/-, none; +, weak; +/++, weak to moderate; ++, moderate.

**Table 5 pmed-1000387-t005:** Evidence of benefits associated with PACS.

Risks				
Review ID	Film Persistence	Record Loss	Increased Time	Increased Costs
Anderson 1997	+/-	+/-	+/-	-
Charvet-Protat 1998	N/A	N/A	+/-	-
Clamp 2005	N/A	N/A	+/-	-

Evidence of risks: N/A, not assessed; +/-, none; -, weak; -/- -, weak to moderate; --, moderate.

### Supporting Clinical Decision Making

#### Computerised provider (or physician) order entry

CPOE systems are typically used by clinicians to enter, modify, review, and communicate orders; and return results for laboratory tests, radiological images, and referrals (for pharmacy see ePrescribing) [8]. These systems can be integrated within EHRs and/or integrate or interface with CDSSs. They not only integrate orders (similar to EHRs) with patient data and PACS images, but they also have the explicit purpose of electronic transfer of orders and the return of results. The electronic request of orders and return of results is expected to result in organisational efficiency gains and time savings. However, potential risks of these systems include increased time spent on computer-related activity and increased infrastructure costs, thereby decreasing overall organisational efficiency.

We found relatively few reviews on CPOE that were not focused primarily on the ordering of medications, rather than the ordering of laboratory tests and medical images. Within the reviews, we found that what had been empirically evaluated generally mirrored the theorised impacts (Tables 6 and 7). The findings from these reviews indicated weak evidence of an impact on organisational efficiency. Individual efficiency and workload both increased and decreased between providers. Additionally, while the speed at which orders were received led to better preparation and a modest effect on time taken to process and deliver results, it did not affect when the patient or their specimen was made available or when their results were acted upon. Findings supported moderate evidence of an impact on practitioner performance. The provision of relevant information at the time of ordering had a moderate impact on increasing cost-conscious ordering and subsequently on decreasing those orders deemed inappropriate; and following system-generated suggestions led to increased ordering of routine care as well as withdrawal of potentially injurious care. There was however evidence that the use of CPOE had a negative impact on practitioners because of the increased time needed to complete orders by having to enter them into the computer system, or incompatibility between professional routines and those imposed by the new system. Changes in workflows also posed an opportunity cost for collaboration, and the potential exclusion of certain providers from processes. Additionally, workload could either decrease or increase as a result of changes in workflow, which when unaccounted for were dealt with on an ad hoc basis and allowed for the redesignation of responsibilities.

**Table 6 pmed-1000387-t006:** Evidence of benefits associated with CPOE.

Benefits					
Review ID	Resource Utilisation	Indicated Care	Patient Outcomes	Cost Savings	Time Savings
Chaudry 2006	+/+ +	+/++	+/-	+/-	+/-
Garg 2005	+/+ +	+/++	+/-	+	N/A
Georgiou 2007	+/-	+	+/-	+	+/-
Jamal 2009	+	+	+/-	N/A	N/A
Niyazkhani 2009	N/A	+	N/A	N/A	+/+ +
Poissant 2005	N/A	N/A	N/A	N/A	+/+ +
Rothschild 2004	+/+ +	+	+/-	+	+
Shekelle 2006	+/+ +	+/++	+/-	+	+

Evidence of benefits: N/A, not assessed; +/-, none; +, weak; +/++, weak to moderate; ++, moderate.

**Table 7 pmed-1000387-t007:** Evidence of risks associated with CPOE.

Risks				
Review ID	Increased Time	Interruptions	Increased Costs	Workarounds
Chaudry 2006	+/-	N/A	+/-	N/A
Garg 2005	N/A	-	+/-	N/A
Georgiou 2007	-	N/A	+/-	N/A
Jamal 2009	N/A	N/A	N/A	N/A
Niyazkhani 2009	-	--	+/-	-
Poissant 2005	- -	-	N/A	N/A
Rothschild 2004	+/-	N/A	+/-	N/A
Shekelle 2006	+/-	N/A	+/-	N/A

Evidence of risks: N/A, not assessed; +/-, none; -, weak; -/- -, weak to moderate; --, moderate.

#### ePrescribing

ePrescribing refers to clinical information systems that are used by clinicians to enter, modify, review, and output or communicate medication prescriptions. This term thus includes stand-alone CDSSs for prescribing purposes [8]. ePrescribing systems can integrate or interface with EHRs or be an element of a broader CPOE system. Like systems for computerised order entry, those for prescribing also have the explicit purpose of electronic transfer between the prescriber and the pharmacy and are rarely mentioned without decision support functionality [125]. ePrescribing systems should result in similar benefits as CPOE systems, including improvements in organisational efficiency and practitioner performance in relation to prescribing. Furthermore, the direct relationship between the therapeutic nature of prescribing of medications and patient outcomes suggests that better prescribing should lead to improved patient outcomes. Finally, as the prescribing of medications is a potentially larger contributor to risks to patient safety than the ordering of laboratory tests or radiology images, there is greater scope for improvements in patient safety by reducing errors in the prescribing process. On the contrary, a flawed or cumbersome system design (e.g., suboptimal specificity and/or sensitivity) and deployment strategies (e.g., insufficient training) may contribute to errors in prescribing and lead to workarounds, putting patients at risk and resulting in clinician dissatisfaction. Prescribers can also become over-reliant on decision support or overestimate its functionality, resulting in decreased practitioner performance.

ePrescribing was the most commonly studied intervention amongst the included reviews. Consequently, we found multiple papers covering most of the theorised impacts (Tables 8 and 9). Moderate evidence for improved organisational efficiency was indicated by the increased productivity of pharmacists, decreased turnaround time, and more accurate communication between prescribers and pharmacy. However, communications between pharmacists and prescribers, although standardised, were less information rich. Weak-to-moderate evidence was indicated for improved practitioner performance due in most part to increased ordering of corollary care, fewer medication errors, and by more optimal prescribing to some extent translating into improved surrogate patient outcomes. There was however far less evidence for improvements in patient level outcomes as even in the case of medication errors, it was unclear what proportion of these actually resulted in patient harm. There was evidence of disruptions in workflow, opportunity costs for collaboration, introduction of risks to patient safety due to “alert fatigue,” and suboptimal deployment strategies resulting from workarounds; there was also some evidence of erroneous assumptions regarding the availability of decision support functionality.

**Table 8 pmed-1000387-t008:** Evidence of benefits associated with ePrescribing.

Benefits							
Reference ID	Surrogate Outcomes	Guideline Adherence	Safer Prescribing	Communication	Patient Outcomes	Resource/Cost Savings	Time Savings
Ammenwerth 2008	Pot. ADEs +/+ +	N/A	MEs ++	N/A	ADEs +	N/A	N/A
Bryan 2008	+	+	N/A	N/A	+/-	+	N/A
Chatellier 1998	+/++	N/A	++/+	N/A	Death +/-Haemorrhage +/-Thromboembolic events +/-	N/A	N/A
Clamp 2005	++	+	MEs ++	+	ADEs +	+	+
Delpierre 2004	+/-	+	MEs+	N/A	+/-	N/A	N/A
Duriex 2008	+/++	+	+/++	N/A	Death +/-	+/++	N/A
Eslami 2007	+	+/+ +	+/-	N/A	+/-	+	+
Eslami 2008	+	+/+ +	+	+	+/-	+	+
Eslami 2009	+	+	N/A	N/A	+/-	N/A	N/A
Fitzmaurice 1998	+	N/A	+	N/A	+	N/A	N/A
Garg 2005	+/++	+	+/-	N/A	+/-	+/-	N/A
Hider 2002	+/++	+/+ +	+	+/+ +	+	+	N/A
Jamal 2009	+	+/++	+	N/A	+/-	+	N/A
Mitchell 2001	+	N/A	N/A	N/A	+/-	+	+
Mollon 2009	+/++	N/A	N/A	N/A	+/-	+/-	N/A
Niyazkhani 2009	+	N/A	N/A	+/++	N/A	N/A	+/++
Poissant 2005	N/A	N/A	N/A	N/A	N/A	N/A	+/-
Rothschild 2004	+/++	+	MEs ++	N/A	ADEs +	+	+
Schedlbauer 2009	+/++	+	MEs ++	N/A	Renal ADEs+Falls+	+	N/A
Shamliyan 2008	+	N/A	MEs ++	N/A	ADEs +	N/A	N/A
Shekelle 2006	+/+ +	N/A	MEs +	N/A	ADEs +	+/-	N/A
Shiffman 1999	N/A	+/+ +	N/A	N/A	+/-	N/A	N/A
Shojania 2009	+	+	N/A	N/A	+	N/A	N/A
Sintchenko 2007	++	+	N/A	N/A	Death +/-ADEs +	N/A	N/A
Tan 2005	+	N/A	MEs +	N/A	+/-	+/-	+
Van Rosse 2009	+/+ +	N/A	++	+	Death +/-ADEs +/-	N/A	+
Wolfstadt 2008	N/A	N/A	N/A	N/A	ADEs +/-	N/A	N/A
Yourman 2008	+	+	N/A	N/A	+/-	+/-	N/A

Evidence of benefits: N/A, not assessed; +/-, none; +, weak; +/++, weak to moderate; ++, moderate.

**Table 9 pmed-1000387-t009:** Evidence of risks associated with ePrescribing.

Risks			
Reference ID	Patient Harm	Increased Time	Increased Costs
Ammenwerth 2008	+/-	N/A	N/A
Bryan 2008	+/-	N/A	N/A
Chatellier 1998	+/-	N/A	N/A
Clamp 2005	+/-	-	+/-
Delpierre 2004	+/-	N/A	N/A
Durieux 2008	+/-	N/A	+/-
Eslami 2007	+/-	-/- -	-
Eslami 2008	+/-	-/- -	-
Eslami 2009	+/-	N/A	N/A
Fitzmaurice 1998	N/A	N/A	N/A
Garg 2005	+/-	N/A	N/A
Hider 2002	+/-	N/A	N/A
Jamal 2009	+/-	N/A	N/A
Mitchell 2001	+/-	+/-	+/-
Mollon 2009	+/-	N/A	+/-
Niyazkhani 2009	N/A	-/--	N/A
Poissant 2005	N/A	-/- -	N/A
Rothschild 2004	N/A	+/-	+/-
Schedlbauer 2009	+/-	N/A	+/-
Shamliyan 2008	+/-	N/A	N/A
Shekelle 2006	+/-	N/A	+/-
Shiffman 1999	+/-	N/A	N/A
Shojania 2009	+/-	N/A	N/A
Sintchenko 2007	+/-	N/A	N/A
Tan 2005	N/A	+/-	+/-
Van Rosse 2009	+/-	+/-	N/A
Wolfstadt 2008	N/A	N/A	N/A
Yourman 2008	N/A	N/A	+/-

Evidence of risks: N/A, not assessed; +/-, none; -, weak; -/- -, weak to moderate; --, moderate.

#### Computerised decision support systems

CDSSs are, when used in the context of eHealth technologies, clinical information systems that integrate clinical and demographic patient information to provide support for decision making by clinicians [8]. These systems have highly variable levels of sophistication and configurability with regards to inputs (patient-specific data), knowledge bases, inference mechanisms (logic), and outputs. They issue certain alerts or prompts, which can take either an active (requiring the user to act on them) or passive (popping up without requiring the user to act on them) form. These decision support systems can be integrated or interface with other systems (such as those discussed above), or simply be stand alone.

In principle, the fundamental impact of CDSSs should be improved clinical decision making. This improvement should, in turn, lead to improved practitioner performance in a variety of care activities (e.g., provision of preventive care, diagnosis, disease management) and ways in which these care activities are delivered (e.g., more evidence-based or guideline adherent decisions). These systems should also be able to help address disparities in care by facilitating standardisation, especially when part of an EHR, PACS, CPOE, or ePrescribing system. Improved practitioner performance should result in a variety of beneficial impacts depending on the care activity targeted (e.g., increased immunisation rates, reduced resource utilisation, more timely diagnosis) or better disease control. In addition, if practitioner's performance is directly related to patient outcomes, then these too should improve. The main theorised risks relating to the use of CDSSs include a potential decline in practitioner performance due to deskilling or flawed system design, and related threats to patient safety.

Actual improved practitioner performance rather than just behaviour change in general was supported by only weak evidence (Tables 10 and 11). While most findings were able to demonstrate some degree of behaviour change it did not always translate into the provision of higher quality care. While some subgroups seemed to fare better than others, the evidence was still only modest at best. The most notable of findings were hallmarked by relative consistency across findings and thusly provided moderate evidence. These included increased provision of preventive care measures, disease-specific examinations or measurements, corollary orders to monitor side effects, and the decreased use of unnecessary or redundant care. Efforts at influencing practitioners to change practice patterns to adhere to a certain model of care were however less successful. No evidence was indicated for an impact on patient outcomes outside prescribing; while surrogate outcomes were modestly improved in some cases there was inconsistency across studies.

**Table 10 pmed-1000387-t010:** Evidence of benefits associated with CDSS.

Benefits				
Reference ID	Indicated Care	Guideline Adherence	Surrogate Outcomes	Patient Outcomes
Balas 2004	+	++	+/++	+/-
Bryan 2008	+	+	+	+/-
Chaudhry 2006	+	++	+	+/-
Delpierre 2004	++	+/-	+	+/-
Dexheimer, 2008	++	+	+	+/-
Garg 2005	+/++	++	+/++	+/-
Hayward 2009	+/-	N/A	+/-	+/-
Heselmans 2009	N/A	+/-	+/-	+/-
Jamal 2009	+/++	++	+	+/-
Jerant, 2000	++	+	+	+/-
Montgomery 1998	+	N/A	+	+/-
Randell 2007	+/-	+/-	+/-	+/-
Shekelle 2006	++	++	+/++	+/-
Shiffman 1999	+	+/++	+	+/-
Shojania 2009	+/++	++	**+**	+/-
Sintchenko 2007	+	+	+/++	+/-
Smith 2007	N/A	N/A	+/-	+/-
Tan 2009	+/-	N/A	+/-	+/-

Evidence of benefits: N/A, not assessed; +/-, none; +, weak; +/++, weak to moderate; ++, moderate.

**Table 11:** Evidence of risks associated with CDSS.

Risks		
Reference ID	Practitioner performance	Patient outcomes
Balas 2004	N/A	+/-
Bryan 2008	N/A	+/-
Chaudhry 2006	N/A	+/-
Delpierre 2004	+/-	+/-
Dexheimer, 2008	N/A	+/-
Garg 2005	N/A	+/-
Hayward 2009	N/A	+/-
Heselmans 2009	N/A	N/A
Jamal 2009	+/-	+/-
Jerant, 2000	+/-	+/-
Montgomery 1998	+/-	-
Randell 2007	-	+/-
Shekelle 2006	N/A	+/-
Shiffman 1999	+/-	+/-
Shojania 2009	N/A	+/-
Sintchenko 2007	+/-	+/-
Smith 2007	N/A	+/-
Tan 2009	+/-	+/-

Evidence of risks: N/A, not assessed; +/-, none; -, weak; -/- -, weak to moderate; --, moderate.

## Discussion

Our systematic review of systematic reviews on the impact of eHealth has demonstrated that many of the clinical claims made about the most commonly deployed eHealth technologies cannot be substantiated by the empirical evidence. Overall, the evidence base in support of these technologies is weak and inconsistent, which highlights the need for more considered claims, particularly in relation to the patient-level benefits, associated with these technologies. Also of note is that we found virtually no evidence in support of the cost-effectiveness claims (Tables 2–11) that are frequently being made by policy makers when constructing business cases to raise funding for the large-scale eHealth deployments that are now taking place in many parts of the world [1].

This work is characterised by a number of strengths and limitations, which need to be considered when interpreting this work. Strengths include the multifaceted approach to the identification of systematic reviews and the synthesis of this body of evidence. Juxtaposing the conceptual maps of the fields of quality, safety, and eHealth permitted us to produce a comprehensive framework for assessing the impact of these technologies in an otherwise poorly ordered discipline. In addition, reflecting on methodological considerations and socio-technical factors enabled us to produce an overview that is sensitive to the intricacies of the discipline.

Given the poor indexing of this literature and the fact that our searches were centred on English-language databases, there is the possibility that we may have missed some systematic reviews. Our use of a novel, multimethod approach may be criticised as being less rigorous than a conventional systematic review in that we were not in a position to appraise individual primary studies. These more novel methods of synthesis are less well developed and employed, and therefore less evaluated [126]. The fact that we needed to adapt the instrument used for critical appraisal is another potential limitation. Further, our assumptions about the theoretical benefits expected presumes that the eHealth technologies considered are capable of delivering these and are used in a manner that allows them to do so. Likewise, it could be argued that some of the expected benefits outlined in this overview are assured and perhaps do not therefore require formal evaluation. It is our view, based on the prevailing climate surrounding EHRs and large-scale implementations underway globally, that the claims made about these technologies are subjected to critical review in the light of the empirical evidence. The overlap in reviews and inconsistent use of terminology required us to make judgment calls regarding what reviews, and indeed which included primary studies, pertained to which interventions. Our focus on clinician-orientated information systems being used in predominantly economically developed country settings are further limitations. More patient-oriented technologies such as telehealth care are no less important than those oriented towards professionals. We are currently engaged in follow-on work, which broadens our field of enquiry along these lines [127]–[131]. Finally, our synthesis was limited by critical deficits within the literature, which undermined our efforts to generate a fully reproducible quantitative summary of findings [132].

At the most elementary level, the literature that constitutes the evidence base is poorly referenced within bibliographic databases reflecting the nonstandard usage of terminology and lack of consensus on a taxonomy relating to eHealth technologies [133]–[135]. There were, furthermore, varying degrees of overlap between individual reviews and contradictory findings even amongst reviews of the same primary studies. In addition, we found considerable heterogeneity in the ways in which findings and other aspects relating to the fundamental features of reviews (motivation, objectives, methods, presentation of findings, etc.) from individual papers were presented. This imprecision and nonstandard usage of terminology, as well as the poor quality of reviews, posed additional challenges, both with respect to interpretation of findings from individual reviews and in relation to synthesising the overall body of evidence.

Our greatest cause for concern was the weakness of the evidence base itself. A strong evidence base is characterised by quantity, quality, and consistency. Unfortunately, we found that the eHealth evidence base falls short in all of these respects. In addition, relative to the number of eHealth implementations that have taken place, the number of evaluations is comparatively small. Apart from several barriers and challenges that impede the evaluation of eHealth interventions per se [136]–[141], a number of factors might contribute to evaluative findings going unpublished [142]. Conflict of interests can, in particular, make it difficult to publish negative findings [142], which means that the potential for publication bias should not be underestimated in this discipline [102],[143]. Moreover, published primary research has been repeatedly found to be of poor quality – particularly with regards to outcome measurement and analysis [73],[74],[80],[86],[118]. The highly heterogeneous and complex nature of these interventions makes consistency of findings, even across very similar scenarios, difficult to detect. Our critical appraisal exercise found the same to be true for secondary research. How the included reviews fared with regards to our critical appraisal, merits further comment and will be the subject of a further publication.

Another commonly criticised element of the existing evidence base is its utility [144]. Evaluations have to date largely favoured simplistic approaches, which have provided little insight into why a particular outcome has occurred [145]. Understanding the underlying mechanisms, typically by studying the particular context of the evaluation, is critical for drawing conclusions in relation to causal pathways and effectiveness of eHealth interventions [146]. In addition, evaluations have tended to focus on the benefits with little attention to the risks and costs, which are rarely assessed or rigorously appraised [73],[74],[80],[86],[118]. Consequently, the existing evidence base is often of little utility to decision making in relation to the strategic direction of implementation efforts [144].

A handful of high-profile primary studies demonstrating the greatest evidence of benefit often serve as exemplars of the transformative power of clinical information systems [22]. These often include advanced multifunctional clinical information systems incorporating storage, retrieval, management, decision support, order and results communication, and viewing functionality. Evidence of the beneficial impact of such systems is limited, however, to a few academic clinical centres of excellence where the systems were developed in house, undergoing extensive evaluation with continual improvement, supported by a strong sense of local ownership by their clinical users [31],[56]. The contrast between the success of these systems and the relative failure of much of the wider body of evidence is striking. Clearly, there are important lessons to be learned from these centres of excellence, but the extent to which the results of these primary studies can be generalised beyond their local environment to those institutions procuring “off-the-shelf” systems is questionable. It is encouraging, however, to see evaluations of commercial systems increasingly taking place [55]. A range of factors tend to contribute to the lack of successful implementations of these off-the-shelf systems. In particular, these commercial systems typically have assumptions about work practices embedded within them, which are often not easily transferable to different contexts of use. Additionally, it is not unusual for insufficient time and effort to be devoted to the all-important customisation process [147]. NHS Connecting for Health's difficulties with the implementation of EHRs into hospitals in England is a prime example of the challenges that can ensue if such socio-technical factors are given insufficient attention [148].

Keeping in mind the above, the maturation of evaluation is vital to the success of eHealth [149],[150]. There is some indication that the quality of evaluations is beginning to improve with regards to methodological rigour [74], but there is clearly still considerable scope for improvement [118]. Most of the reviews we included in our work made calls for more rigorous research to establish impact with some calling for more randomised controlled trials (RCTs) in particular [61],[151]. A growing number of authors have however argued for trials of eHealth interventions to employ guidance specifically for complex interventions [152]. However, there are a number of challenges to conducting RCTs of eHealth [153], and many calls have also been made for using other complementary methodologies [24],[146]. Strategies for improving the quality of research should include building the capacity and competency of researchers. In the shorter term, developing resources, tool-kits, frameworks, and the like for researchers and consumers of research should be prioritised [154]–[156]. Such developments are pivotal to furthering the science of evaluation in eHealth and the use of evidence-based principles in health informatics [157]. Another important development that is needed is the collaboration of different disciplines in evaluation [158],[159].

We found an important literature pertaining to the design and deployment aspects of eHealth technologies. This literature is central to understanding why some interventions succeed and others fail (or being judged as such). At the individual level, “human factors” play an important role in the design of an intervention, determining usability and ultimately adoption [160]. At the aggregate level, “organisational issues” are critical in strategising deployment that ultimately influences adoption [160]. Although both enablers and barriers to success are being elicited retrospectively from the literature for design, development, and deployment, the findings for both of these concepts, inter-related as they are, have largely gone untested prospectively. Although there is greater attention being paid to the socio-technical aspects in formal evaluations than ever before, there is still much that needs to be understood [161].

### Conclusions

It is clear that there is now a large volume of work studying the impact of eHealth on the quality and safety of health care. This might be seen as setting a firm foundation for realising the potential benefits of eHealth. However, although seminal reports on quality and safety of health care invariably point to eHealth as one of the main vehicles for driving forwards sweeping improvements [2]–[7], our work indicates that realising these benefits is not guaranteed and if it is to be achieved, this will require substantial research resources and effort.

Our major finding from reviewing the literature is that empirical evidence for the beneficial impact of most eHealth technologies is often absent or, at best, only modest. While absence of evidence does not equate with evidence of ineffectiveness, reports of negative consequences indicate that evaluation of risks – anticipated or otherwise – is essential. Clinical informatics should be no less concerned with safety and efficacy than the pharmaceutical industry. Given this, there is a pressing need for further evaluations before substantial sums of money are committed to large-scale national deployments under the auspices of improving health care quality and/or safety.

Promising technologies, unless properly evaluated with results fed back into development, might not “mature” to the extent that is needed to realise their potential when deployed in everyday clinical settings. The paradox is that while the number of eHealth technologies in health care is growing, we still have insufficient understanding of how and why such interventions do or do not work [123]. To resolve this, it is essential to not only devote more effort to evaluation, but to ensure that the methodology adopted is multidisciplinary and thus capable of untangling the often complex web of factors that may influence the results. Moreover, a fuller description of the rationale for the choice of methodological approach employed to evaluate eHealth technologies in health care would facilitate synthesis and comparison.

Finally, it is equally important that deployments already commissioned are subject to rigorous, multidisciplinary, and independent evaluations. In particular, we should take every opportunity to learn from the largest eHealth commissioning and deployment project in health care in the world – the £12.8 billion NPfIT and the at least equally ambitious national programme that has recently begun in the US [162]–[166]. These and similar initiatives being pursued in other parts of the world offer an unparalleled opportunity not just for improving health care systems, but also for learning how to (or how not to) implement eHealth systems and for refining these further once introduced.

## Supporting Information

Table S1 Critical appraisal form.
(0.05 MB DOC)

Table S2 Characteristics and main findings of “reviews.”
(0.42 MB DOC)

Text S1 Search strategy (databases, string, and filters).
(0.05 MB DOC)

Text S2 Intervention inclusion and exclusion criteria.
(0.03 MB DOC)

## Abbreviations

CDSS: computerised decision support system
CPOE: computerised provider (or physician) order entry
EHR: electronic health record
ePrescribing: electronic prescribing
NHS: National Health Service
PACS: picture archiving and communication system
