# Supplementary material for: The Impact of eHealth on the Quality and Safety of Health Care: A Systematic Overview
Source: PLoS Med. 2011 Jan 18;8(1):e1000387. doi: 10.1371/journal.pmed.1000387 (PMC3022523; doi:10.1371/journal.pmed.1000387)
Supplement: Table S1 — Critical appraisal form. (0.05 MB DOC) [file pmed.1000387.s001.doc]

**Table S1: Critical appraisal form**

Adapted from:

Critical Appraisal Skills Programme (CASP), Public Health Resource Unit, Institute of Health Science, Oxford.

Oxman AD, Cook DJ, Guyatt GH (1994). Users’ guides to the medical literature. VI. How to use an overview. JAMA 272: 1367-1371.

| REVIEW FOCUS | Clearly (+2) | Somewhat (+1) | No or can’t tell (0) |
| --- | --- | --- | --- |
| 1. Did the review address a clearly focussed issue?  Consider whether the authors reported inclusion criteria according to PICO or whether there was substantial variation in the studies included with regards to:   - Participants (end-users and patients) - Settings - Domain (condition, activity, impact and outcomes considered) - Comparisons made |  |  |  |
| 2. Did the review assess a clearly focussed technology?  Consider whether the authors reported explicit inclusion criteria regarding the technical specification of the intervention and its use or whether there was substantial variation in the studies regarding the intervention and its use.  If more than one technology was assessed, were the technologies and their relationship to the other technologies clearly delineated? |  |  |  |
| 3. Did the authors look for the appropriate sort of papers?  Consider whether the authors reported what study designs were eligible and the reasons for doing so. Additionally, consider whether the studies had:   - Outcomes relevant to review objective - A design and methods appropriate for addressing the review objective |  |  |  |

| VALIDITY OF REVIEW RESULTS | Clearly (+2) | Somewhat (+1) | No or can’t tell (0) |
| --- | --- | --- | --- |
| 4. Do you think the important, relevant studies were included?  Look for the usual on the databases used, whether unpublished research searched, if relevant references reviewed, plus searching:   - Health Informatics resources - Personal contact with experts - Internet   Additionally, does the search string demonstrate awareness of indexing issues in HI? Has the review been update if much time has passed since the searches and publication? |  |  |  |
| 5. Did the review’s authors do enough to assess the quality of the included studies?  Consider whether they performed assessment with tool , characterised quality by assigning level of evidence by study type only, or limited inclusion to rigorous designs etc (unit of analysis especially important, contamination, confound). |  |  |  |
| 6. Were the studies accurately described?  Look for the usual on design, methods and results and quality plus system functionality, integration, deployment strategy, user-system interaction, organisational context, timing of study, and any negative findings. |  |  |  |
| 7. Are the results reported in a clear and meaningful way?  How are the results structured and how are they presented (summarisation, reproduction or interpretation, neither) |  |  |  |
| 8. If the results of included have been combined, was it reasonable to do so?  Consider if there was substantial heterogeneity amongst studies in terms of settings and/or conditions, quality, comparisons made, timing of, system-user interaction, sophistication and/or and deployment strategy.  For meta-analysis consider in addition the choice of effects model and summary measures, and the results of interaction or statistical heterogeneity.  Finally whether the reasons for any variations in impact are analysed and/or discussed. |  |  |  |
| 9. Did the review demonstrate awareness of its own limitations?  Consider whether the review noted   - Limitations of included studies - Limitations of the review itself - Findings in light of research |  |  |  |

| RESULTS | Clearly (+2) | Somewhat (+1) | No or can’t tell (0) |
| --- | --- | --- | --- |
| 10. Does the review present an overall result?  Consider   - If you are clear about the reviews ‘bottom line’ results - What these are (numerically or narratively) - How were the results expressed (conclusiveness/doubt) |  |  |  |
| 11. How precise are the results?  Are the results presented with confidence intervals if expressed numerically? Narrative structure for assigning levels of evidence? |  |  |  |

| APPLICABILTY | Clearly (+2) | Somewhat (+1) | No or can’t tell (0) |
| --- | --- | --- | --- |
| 12. Next steps or implications for considering implementation of such technologies? Appropriate based on findings and other pertinent factors? Further research indicated? |  |  |  |
| 13. Are the results generalisable beyond the confines of the setting in which the work was originally conducted?  Consider whether the authors noted the generalisability of their results and any heterogeneity. Additionally, consider whether enough detail was presented on system functionality, integration, deployment strategy, user-system interaction, organisational context, timing of study, and any negative findings. Importantly, the proportion of studies from benchmark leaders and academic institutions should also be considered. |  |  |  |
| 14. Were all relevant outcomes considered?  Depending on the review objective, were all outcomes relevant to answering that question considered? |  |  |  |
| 15. Are you able to assess the benefit versus risk and costs?  Even if this is not addressed by the review, what do you think? Was there any discussion of negatives such as costs or risks? |  |  |  |
