# Supplementary material for: The Impact of eHealth on the Quality and Safety of Health Care: A Systematic Overview
Source: PLoS Med. 2011 Jan 18;8(1):e1000387. doi: 10.1371/journal.pmed.1000387 (PMC3022523; doi:10.1371/journal.pmed.1000387)
Supplement: Table S2 — Characteristics and main findings of “reviews.” (0.42 MB DOC) [file pmed.1000387.s002.doc]

**Table S2: Characteristics and main findings of “reviews”**

| **Reference & topic** Ammenwerth 2008 – Impact of eRx /CDSS on medication safety | | **Critical appraisal score**  24 |
| --- | --- | --- |
| **Studies and interventions**  27 RCTs, CTs, CBAs, and ITSs – 1996 to 2006  Majority in US in normal inpatients  Commercial and home-grown systems, advanced and basic/intermediate decision support, most decision support non drug-specific | **Limitations of studies**  Validity: accounting for clustering, blinding, control for confounding, design, detection bias, methodological reporting (inclusion/exclusion criteria, baseline differences and adjustments, treatment during study, missing values, follow-up, instrument validity), selection of controls  Utility: sufficient details on intervention and context provided | **Limitations of review**  Heterogeneity: metrics, study designs, systems  Generalisability: home-grown systems, limited sites  Validity: retrieval, publication bias, selection bias, inter-study dependence, metrics, sub-groups  Scope: long-term effects, negative impact |
| **Findings on impact**  Twenty-three of 25 studies analysing effect on the ME rate showed a significant relative risk reduction of 13% to 99%. Six of the nine studies analysing effect on potential ADEs showed a significant relative risk reduction of 35% to 98%. Four of the seven studies analysing effect on ADEs showed a significant relative risk reduction of 30% to 84%. Findings indicate that eRx can substantially reduce the risk for MEs and potential and actual ADEs. There is especially good evidence for a positive effect of eRx with advanced decision support in hospital settings. | **Findings on moderators of effect**  Sub-group analysis indicated a higher relative risk reduction by home-grown systems relative to commercial; eRx compared with handwritten orders relative to advanced decision support compared to limited; advanced decision support relative to limited or none; and manual chart review to automatic database analysis of prescriptions.  Other graphical sub-group analyses did not indicate differences between level of care (normal or intensive), patient groups (elderly, children, unspecific), type of drugs (specific or unspecific), or study design (BA, CT, RCT). | **Further research indicated**  RCTs, across multiple sites, in different settings, commercial systems, primary care, outside US, minimising bias,  Standardised reporting for health informatics evaluations    Multi-disciplinary methods  Socio-technical factors on barriers/success  Evaluation of long-term effects |

| **Reference & topic** Anderson 1997 – Impact of PACS on practitioner performance, patient outcomes, organisational efficiency and costs | | **Critical appraisal score**  23 |
| --- | --- | --- |
| **Studies and interventions**  22 studies of variable design – 1990 to 1997 | **Limitations of studies**  Validity: design, power  Scope: inclusion of relevant processes/outcomes in evaluation | **Limitations of review**  Quantity: economic analyses, organisational efficiency |
| **Findings on impact**  Based on the available studies and the application of quality criteria, there has not yet been a definitive demonstration that digital imaging, viewed either on a workstation or printed to film, is equivalent to analog film for making a primary diagnosis of all of the clinical conditions that present in a varied patient population.  Data suggest that PACS are more efficient at retrieving both current and archived images, as well as generating and delivering images than film-based systems. Equivalence in interpretation time and time till examination indicated.  It remains to be demonstrated that overall clinical and production processes are more efficient, or that those efficiencies translate into improved quality, increased access, or reduced costs of care. | **Findings on moderators of effect**  Inexperience in reading images on workstations and unwillingness to use workstations interactively to improve the quality of the imaging were factors discussed relating to poorer diagnostic accuracy with PACS. Use of image processing software tools were related to increased interpretation time with PACS. | **Further research indicated**  Internally valid. macro effects on organisational efficiency, cost-savings and cost-benefit  Multi-disciplinary methods |

| **Reference & topic**  Balas 2004 – Impact of CDSS for diabetes care on practitioner performance | | **Critical appraisal score**  22 |
| --- | --- | --- |
| **Studies and interventions**  9 RCTs – 1976 to 1997  Mostly in the US  Many from benchmark institutions | **Limitations of studies**  Validity: power | **Limitations of review**  Validity: publication bias |
| **Findings on impact**  The primary intervention in these nine studies was aimed at physicians and consisted of clinically relevant and diabetes guideline-based prompts. Overall compliance with recommended diabetes care procedures was 71% to 227% higher in the prompted group than those in the control group. Compliance with diabetes care guidelines was also significantly better (p≤0.05) among the intervention group than in the control group (e.g. higher routine diabetes care visit rates per patient during the study period, glycated hemoglobin determinations, eye and foot examinations, and compliance with suggested test ordering and other diabetes care procedures). Yet, there were also particular outcomes for which there was not a significant difference between the intervention and the control group (e.g. HgbA1c assessment compliance, foot and ophthalmologic examination compliance, HgbA1c and fasting blood sugar compliance, self-measurement of blood glucose compliance, and referral to dietary clinic). Four of the studies provided a general measure of compliance to care measures. In these studies, the overall adherence score was calculated by dividing the number of items completed in accordance with the guidelines by the total number of items recommended for the patient. Three of the four studies showed significant improvement (p≤0.05) in the overall adherence measure. Computerised prompting to healthcare professionals appears to have an impressive impact on the compliance with recommended diabetes care guidelines and procedures. | **Findings on moderators of effect** | **Further research indicated**  Trials testing clinical performance and patient outcomes  Economic analyses, long-term effects |

| **Reference & topic** Bennet 2003 – Impact of eRx/CDSS on practitioner performance | | **Critical appraisal score**  18 |
| --- | --- | --- |
| **Studies and interventions**  26 RCTs – 1976 to 2001  Majority in US outpatient care settings in academic affiliates  Many from benchmark institutions  Either feedback or reminders provided | **Limitations of studies**  Validity: baseline differences and adjustments, follow-up, unit of analysis | **Limitations of review**  Heterogeneity: systems, metrics, settings, participants  Generalisability: home-grown systems, academic affiliates |
| **Findings on impact**  Reminders (patient-specific, synchronous) to providers in outpatient settings generally improved medication management with 6 of 12 comparisons showing improvement (RRs, 1.0 to 42). Physician feedback (aggregative, asynchronous) in outpatient settings generally had smaller effects than reminders with five of seven comparisons showing improvement (RRs, 1.0 to 2.5). There were mixed results from feedback aimed at containing prescribing costs. Reminders to providers in inpatient settings generally improved with three of five comparisons showing improvement (RRs, 1.0 to 2.1). | **Findings on moderators of effect**  The reminder system was considered to be easy to implement elsewhere in 69% of cases. Poor user-interface and redundant data entry were cited as reasons for poor system utilisation.  The results support the contention that information is most effective if presented close to the time of decision making, reminders being synchronous compared with asynchronous feedback. | **Further research indicated**  Technical specification effects  HFs on barriers/success |

| **Reference & topic**  Bryan 2008 – Impact of CDSS on practitioner performance and patient outcomes in the primary care setting | | **Critical appraisal score**  19 |
| --- | --- | --- |
| **Studies and interventions**  17 RCTs, CTs and observational studies – 2000 to 2006  Majority in US, split between academic and community settings  Majority active systems embedded within EHR delivered at the point of care for of chronic disease management | **Limitations of studies**  Validity: allocation concealment, baseline differences and adjustments, consistency of intervention use, control of confounding, power, selection bias | **Limitations of review**  Generalisability: older systems, academic affiliates, long-term effects  Heterogeneity: systems, user-system interaction |
| **Findings on impact**  Overall, 76% of studies had either partial or complete improvement in outcomes documented. Nine of the studies found definitive positive outcomes an additional four studies showing improvement for some of the outcomes measured. No study demonstrated a negative finding finding (patient harm or deterioration related to the intervention). This work reinforced previous work by showing positive correlation between the use of decision support in the outpatient setting and improved outcomes. | **Findings on moderators of effect**  Studies with neutral findings evaluated systems with either of limited functionality or easily side-stepped. | **Further research indicated**  Internally valid, outpatient settings, across multiple sites and varied settings, newer systems, chronic disease management systems, patient outcomes  HFs and socio-technical issues for barrier/success |

| **Reference & topic**  Charvet-Protat 1999 – Costs and benefits of PACS | | **Critical appraisal score**  12 |
| --- | --- | --- |
| **Studies and interventions**  17 comparative studies – 1990 to 1996 | **Limitations of studies**  Validity: design  Scope: inclusion of relevant processes/outcomes in evaluation | **Limitations of review**  Generalisability: immature systems, costs |
| **Findings on impact**  The economic evaluation comparing PACS and film-based systems shows an increased in cost for PACS. Savings related to PACS (films, archive space, staff) are currently insufficient to off-set the cost of the initial investment and service contracts. It would appear that five to six years are needed before the savings generated by PACS even-out the initial investment. Advantages such as: faster medical decision making, improved patient relations, lower mean hospital stay and better management of archives have been suggested and sometimes observed. | **Findings on moderators of effect**  A notion of threshold of activity has been introduced by several authors. Indeed, two studies reported that when the volume of examinations stored on PACS is low, the savings generated by the system are less and do not offset the initial investment**.** | **Further research indicated**  New evaluation techniques to counter the non-generalisable problem of existing structure and patient-mix |

| **Reference & topic** Chatellier 1998 – Impact of eRx/CDSS for anticoagulant management on practitioner performance and patient outcomes | | **Critical appraisal score**  21 |
| --- | --- | --- |
| **Studies and interventions**  9 RCTs – 1987 to 1997  Most on oral anticoagulation therapy with warfarin | **Limitations of studies**  Validity: power | **Limitations of review**  Validity: retrieval bias, publication bias, metrics, unit of analysis, power, sub-groups, fixed-effects |
| **Findings on impact**  Meta-analysis of seven studies found eRx for anticoagulation therapy to increase the proportion of visits where patients were within the therapeutic range by 29% (global OR 1.29, 95%CI 1.12 to 1.49) there was however significant heterogeneity (p=.02) between trials. Excluding one of the smallest studies and which provided the largest effect changed the global OR to 1.25 (95%CI 1.08 to 1.45) with non-significant heterogeneity (p=.12). For both heparin and warfarin, the therapeutic range was attained more quickly in the patients in the computer group than in the controls group Moreover, the computer group spent more time within the therapeutic range for both inpatients and outpatients.    In the studies where data were available, the proportion of patients under-treated was lower in the computer group than in the control group, whereas the proportion of patients over-treated was higher in the computer group than in the control group in two of three studies. In the six studies where patient outcomes were recorded there was a tendency towards a decreased risk of hemorrhage and a slightly higher risk of thrombotic event in the computer groups compared with the control groups. | **Findings on moderators of effect** | **Further research indicated**  Evaluating usability, cost-effectiveness in usual healthcare settings  Clinical net benefit assessed in a population of patients at various risk of thrombotic events, through a clinical trial of appropriate power  Comparing against the use of a prothrombin time monitor by patients |

| **Reference & topic** Chaudhry 2006 – Impact of HIT systems on practitioner performance, patient outcomes, medication safety, organisational efficiency and costs | | **Critical appraisal score**  18 |
| --- | --- | --- |
| **Studies and interventions**  74 studies of variable design – 1976 to 2005  All in US, most in academic affiliates with majority from benchmark institutions  All multi-functional systems that included electronic documentation or order entry capabilities, majority included decision support, majority home-grown | **Limitations of studies**  Validity: design, selection of controls  Scope: inclusion of relevant processes/outcomes in evaluation  Utility: sufficient details on intervention and context provided | **Limitations of review**  Quantity: quantitative and qualitative studies on implementation, economic analyses, interoperability  Heterogeneity: systems  Generalisability: home-grown systems |
| **Findings on impact**  Studies from four benchmark institutions demonstrate that implementing a multi-functional system can yield real benefits in terms of increased delivery of care based on guidelines (particularly in preventive care), enhanced monitoring and surveillance activities, reduction of MEs, and decreased rates of utilisation for potentially redundant or inappropriate care.  Data on commercial systems, in general, support the findings  of studies from the benchmark institutions on the effect of HIT systems in reducing utilisation and ME. However, they do not support the findings of increased adherence to protocol-based care. In contrast to all previous studies on eRx systems, one study used a mixed quantitative–qualitative approach to investigate the possible role of such a system in facilitating PEs. Twenty-two types of ME risks were found to be facilitated by eRx, relating to two basic causes: fragmentation of data and flaws in user-system interface. | **Findings on moderators of effect**  A disproportionate amount of literature on the benefits that have been realised comes from a small set of early-adopter institutions that implemented home-grown HIT systems. These institutions had considerable expertise in health informatics and implemented systems through incremental development over many years of an internally designed system led by academic research champions. | **Further research indicated**  Standardised reporting for health informatics evaluations  Commercial systems in community settings with relevant economic analyses  Data on initial capital costs, effect on provider productivity, resources required for staff training (such as time and skills), and workflow redesign  HFs and socio-technical issues and barriers/success |

| **Reference & topic** Clamp 2002 – Impact of HIT systems on practitioner performance, patient outcomes, medication safety, organisational efficiency and costs | | **Critical appraisal score**  17 |
| --- | --- | --- |
| **Studies and interventions**  Over 70 studies of variable design – 1997 to 2005  Many from benchmark institutions | **Limitations of studies**  Validity: Hawthorne effect, selection of controls  Scope: inclusion of relevant processes/outcomes in evaluation  Utility: sufficient details on intervention and context provided | **Limitations of review**  Quantity: macro effects, patient outcomes  Generalisability: idiosyncratic sites, involvement of authors in development and evaluation, specialist settings |
| **Findings on impact**  There is positive evidence of process change associated with the EHR. Equally, there is also evidence that EHR can increase time-costs, particularly for physicians. More generally, the evidence presented is quite mixed, in the sense that gains have to be balanced against losses. For example, a reduction in nursing staff time spent on handling document-based tasks has to be balanced against an increase in physician or pharmacist time. As a result, some of the observations are not straightforward – not negative, but not unequivocally positive either. In many papers the evidence was not decisive. There is, for example, evidence of clinician dissatisfaction with EHR, and also evidence that little of the available functionality is used. There is no compelling evidence that EHR reduce the incidence of ADEs, or that the introduction of EHR increases – or decreases – consultation time. It is for all practical purposes true to say that we found no technically sound evidence about cost changes associated with EHR, bar a paper on PACS. Similarly, we found limited evidence about the impact of EHR on patient experiences and outcomes. We have found no evidence at all about “network effects”. The papers we identified tend to support two theories of change. They support the view that EHR can have a direct impact on behaviour, and that it can influence communication patterns and the quality of communications (generally positively). We did not find studies which shed any light on any other possible mechanisms whereby EHR influence behaviour at the “micro” level. | **Findings on moderators of effect**  None noted | **Further research indicated**  None noted explicitly |

| **Reference & topic** Delpierre 2004 – Impact EHR on practitioner performance and patient outcomes | | **Critical appraisal score**  13 |
| --- | --- | --- |
| **Studies and interventions**  25 studies of variable design – 2000 to 2003  Split between commercial and home-grown systems  Variable settings and intervention targets | **Limitations of studies**  Validity: design  Utility: sufficient details on intervention and context provided | **Limitations of review**  Validity: publication bias |
| **Findings on impact**  Six studies analysed the impact of EHRs on the length of the consultation. An increase in this length was observed in three studies. EHRs were also found to change the content of the consultation in the three studies in which it was analysed. Five of eight studies found an increase of guideline adherence. In studies of arterial hypertension and major depression there was no improvement in medical practice and compliance with guidelines. All three studies which analysed preventive care measures demonstrated increased compliance. Only four of nine studies demonstrated improved prescribing practice. One of five studies was able to demonstrate an improved surrogate patient outcome.  A clear positive impact on preventive care was noted. This finding is consistent with other systematic reviews. Improvement in medical practice and the adoption of guidelines was less certain. Positive experiences were as frequent as experiences showing no benefit. Studies evaluating impact on patient outcomes showed no benefit. | **Findings on moderators of effect**  The EHR was perceived as a physical barrier that could have a negative impact on the patient-provider relationship, particularly by reducing eye contact. Concerns were expressed about data confidentiality, personal and professional privacy, bug management, and the additional work for physicians. Factors influencing perception of the system were its characteristics, such as its interface and ease of use, as well as users’ characteristics. Perception of the EHR was better for community-based physicians than for academic-based physicians. Physicians accustomed to working with a computer had a better perception of EHRs. In another study, reduced administrative work and increased accessibility to care protocols, especially for young nurses, were reported. Negative points were the lack of flexibility of these tools, the loss of nurses’ judgment, and the additional work-load. | **Further research indicated**  Standardised reporting for health informatics evaluations  Multi-disciplinary methods  Technical specification effects  HFs and socio-technical issues on barriers/success |

| **Reference & topic** Dexheimer 2008 – Impact of CDSS for preventive care on practitioner performance | | **Critical appraisal score**  19 |
| --- | --- | --- |
| **Studies and interventions**  42 RCTs – 1976 to 2004  Majority in US primary care settings in academic affiliates  Both computerised and computer-generated prompts | **Limitations of studies**  Scope: inclusion of relevant processes/outcomes in evaluation  Utility: sufficient details on intervention and context provided | **Limitations of review**  Generalisability: clinicians  Validity: control of confounding, publication bias, metrics |
| **Findings on impact**  Computer-generated reminders were implemented 136 times and had a mean increase of 12% (Range, -24 to 59; SD, 13). Computerised reminders were employed in 48 interventions and resulted in a 13% mean increase (Range, 8 to 60; SD, 18). | **Findings on moderators of effect** | **Further research indicated**  Socio-technical issues |

| **Reference & topic** Durieux 2008 – Impact of eRx /CDSS for drug dosing on practitioner performance and patient outcomes | | **Critical appraisal score**  23 |
| --- | --- | --- |
| **Studies and interventions**  23 RCTs and CTs – 1984 to 2002  Split between US and Europe and inpatient and outpatient settings | **Limitations of studies**  Validity: allocation concealment, baseline differences and adjustments, contamination, follow-up, power, unit of allocation  Utility: sufficient details on intervention and context provided | **Limitations of review**  Quantity:  Heterogeneity: study settings, outcomes,  Generalisability: limited drugs, older drugs, specialist settings  Validity: control of confounding |
| **Findings on impact**  In summary, computerised advice seems to lead to a change in the initial dose of drug. No effect on the maintenance dose and the total amount of drugs used nor did it reduce the number of dosage adjustments. The results tend to suggest that computerised advice leads to higher serum concentrations, more rapid therapeutic stabilisation and reduces the risk of toxic drug level. These results are in line with the initial review. No difference was observed between the computer and control groups for death rates or for adverse reactions. The initial review however showed significant benefits on adverse reactions. | **Findings on moderators of effect**  In addition, we did not find strong evidence of any logistic and organisation of care aspects favouring the effect of computer support. | **Further research indicated**  Cluster RCTs  Technical specification effects  HFs and STI for barriers/success  Drugs with a narrow therapeutic window or complicated pharmacokinetics |

| **Reference & topic** Eslami 2007 – Impact of eRx w/o CDSS on practitioner performance, patient outcomes, medication safety and organisational efficiency in the outpatient setting | | **Critical appraisal score**  18 |
| --- | --- | --- |
| **Studies and interventions**  30 CTs and observational studies – 1984 to 2006 | **Limitations of studies**  Validity: design  Scope: inclusion of relevant processes/outcomes in evaluation | **Limitations of review**  Quantity: ADE/ME  Validity: retrieval bias, control for confounding |
| **Findings on impact**  In spite of the cited merits of enhancing safety and reducing costs, published evaluation studies do not provide adequate evidence that eRx provides these benefits in outpatient settings. In contrast, there is more evidence on the ability of eRx to increase guideline adherence in outpatient settings. None of the included studies documented the introduction of new errors. The results obtained by non-randomised studies were more likely to report statistically significant improvement in the outcome measures than RCTs. | **Findings on moderators of effect**  Studies on alerts show that alerts were largely ignored by physicians. There was a wide variability in the degree of eRx usage with 2.8% to 90% of all prescriptions entered electronically across four studies and another showing that the levels of system usage were low. However, another study showed there was continued improvement in system usage and usability. During prescribing, physicians were more willing to use passive decision support as the clinical situation became more complex while for simple cases the reminder-based active decision support was more appropriate. | **Further research indicated**  Standardised reporting for health informatics evaluations  Outpatient sub-groups, specific drugs  Technical specification effects  HFs for barriers/success  Multi-disciplinary methods |

| **Reference & topic** Eslami 2008 – Impact of eRx w/o CDSS on practitioner performance, patient outcomes, medication safety and organisational efficiency in the inpatient setting | | **Critical appraisal score**  18 |
| --- | --- | --- |
| **Studies and interventions**  67 RCTs, BAs and observational studies – 1989 to 2006  Most in US  Many from benchmark institutions  Most employed relatively little decision support | **Limitations of studies**  Validity: design, power  Utility: sufficient details on intervention and context provided | **Limitations of review**  Heterogeneity: study designs and settings, intervention, implementation strategy, nature and types of medication and treatment  Generalisability: system-context dependence  Validity: publication bias |
| **Findings on impact**  The impact of eRx systems was especially positive in the category adherence to guidelines, but also to some extent in alerts and appropriateness of alerts; costs and organisational efficiency; and satisfaction and usability. Although on average, there seems to be a positive effect of eRx on safety, studies tended to be non-randomised and were focused on ME rates, and not powered to detect a difference in ADE rates. Some recent studies suggested that errors, ADEs and even mortality increased after CPOE implementation. Only in the category time the impact has been shown to be negative, but this only refers to the physician’s time, not the net time. Except for safety, on the whole spectrum of outcomes, results of RCT studies were in line with non-RCT study results. | **Findings on moderators of effect**  Studies found that most of the alerts were ignored by physicians and when alerts were classified as high level they were accepted more often than those classified as low.    [Quantitative studies show high adherence to alerts. However qualitative studies show many over-ridden alerts. Acceptance rate increase with the clinical importance of the alerts.] | **Further research indicated**  Powerful, internally valid trials, with more decision support for specific patient groups, high risk drugs, and typical ADEs    Multi-disciplinary methods  Technical specification effect  HFs and socio-technical issues on barriers/success |

| **Reference & topic** Eslami 2009 – Impact of eRx/CDSS for glucose regulation on practitioner performance and patient outcomes in the intensive/critical care setting | | **Critical appraisal score**  16 |
| --- | --- | --- |
| **Studies and interventions**  17 RCTs, CBAs and observational studies – 2005 to 2008  Majority used by nurses, stand-alone systems with manual data entry in critiquing mode | **Limitations of studies**  Validity: baseline differences and adjustments, control for confounding  Utility: sufficient details on intervention and context provided | **Limitations of review**  Heterogeneity: case-mix, insulin therapy, associated therapies, systems, metrics |
| **Findings on impact**  All controlled studies reported on at least one quality indicator that was improved by introducing the CDSS (with or without the new protocol). Among controlled studies, one study reported that the number of hypoglycemia events increased but without mentioning whether this increase was statistically significant. Among other studies controlled studies one study did not report, six studies reported no change, and two studies even reported a reduction in the number of hypoglycemia events. Seven observational studies also reported that the number of hypoglycaemic events was in an acceptable range. No clinical symptoms attributable to hypoglycemia were reported in any of the studies. | **Findings on moderators of effect** | **Further research indicated**  Standardised metrics and reporting for glycaemic control  Standardised reporting for health informatics evaluations  HFs for barriers/success |

| **Reference & topic** Fitzmaurice 1998 – Impact of eRx/CDSS for oral anticoagulation management on practitioner performance in the ambulatory care setting | | **Critical appraisal score**  12 |
| --- | --- | --- |
| **Studies and interventions**  7 studies of unknown methodology – 1984 to 1996  Majority in primary care settings  Three systems only, for warfarin only | **Limitations of studies**  None noted explicitly | **Limitations of review**  None noted |
| **Findings on impact**  Evidence from one study that eRx/CDSS can achieve improved therapeutic control in terms of INR, when compared to human performance, Since publication there have several other studies which add to the body of evidence. One study, based in primary care, reported significant improvement in patient control compared with previous hospital-based management. | **Findings on moderators of effect**  None noted | **Further research indicated**  Full performance evaluation in field to be used |

| **Reference & topic** Garg 2005 – Impact of CDSS on practitioner performance and patient outcomes | | **Critical appraisal score**  20 |
| --- | --- | --- |
| **Studies and interventions**  100 RCTs and CTs – 1973 to 2004  Majority in US outpatient settings with academic affiliation  Majority of active systems, suggesting new orders at point of care  Both computerised and computer-generated output | **Limitations of studies**  Validity: allocation concealment, metrics, power, randomisation process | **Limitations of review**  Heterogeneity: systems, outcomes  Generalisability: home-grown systems, academic affiliates  Validity: inclusion/exclusion criteria, Hawthorne effect, sub-groups, summary measure/metrics  Evaluation of the clinical appropriateness of either the knowledge base, algorithms or output |
| **Findings on impact**  Practitioner performance improved in the majority (64%) of studies. The CDSS improved practitioner performance in 62 of 97 (64%) of 97 studies assessing this outcome, including 4 of 10 diagnostic systems, 16 of 21 reminder systems, 23 of 37 disease management systems, and 19 of 29 drug dosing or prescribing systems. Fifty-two studies assessed one or more patient outcomes, of which seven studies (13%) reported improvements. Only 13% of trials evaluated the impact of the decision support on clinician work-flow, with more than half requiring more time and effort from the user compared with paper-based methods. | **Findings on moderators of effect**  Improved practitioner performance was associated with active decision support compared with passive systems (success in 73% of studies vs 47%; p=.02) and studies in which the authors also developed the decision support software compared with studies in which the authors were not the developers (74% success vs 28%, respectively; p<.001). No other predefined study-level co-variate was associated with success. | **Further research indicated**  Effect on patient outcomes |

| **Reference & topic** Georgiou 2007– Impact of CPOE for the ordering of pathology tests on practitioner performance in the inpatient setting | | **Critical appraisal score**  21 |
| --- | --- | --- |
| **Studies and interventions**  19 RCTs, CTs, CBAs and ITS – 1990 to 2004  Majority in North America  Many from benchmark institutions | **Limitations of studies**  Validity methodological reporting  Scope: inclusion of relevant processes/outcomes in evaluation  Utility: sufficient details on intervention and context provided | **Limitations of review**  Quantity: pathology work-processes, turnaround times, test processing stage  Heterogeneity: study designs  Generalisability: older systems, home-grown, academic affiliates, specialist settings |
| **Findings on impact**  There are data suggesting that CPOE is beneficial for clinical and laboratory work process. Few data however are available regarding the impact of CPOE on patient outcomes. | **Findings on moderators of effect**  None noted | **Further research indicated**  Impact on clinical and laboratory work-processes  Comprehensive economic evaluation of immediate  and long-term effects |

| **Reference & topic** Hayward 2009 – Impact of eRx/CDSS for drug monitoring/medication safety in the ambulatory setting | | **Critical appraisal score**  14 |
| --- | --- | --- |
| **Studies and interventions**  7 CTs and observational studies – 2003 to 2008  Majority in HMOs | **Limitations of studies**  Utility: sufficient details on intervention and context provided | **Limitations of review**  Quantity: patient outcomes, economic analyses, HIT interventions to improve drug monitoring  Heterogeneity: drug sets monitored, phase of drugs, time of alert  Validity: publication bias, critical appraisal |
| **Findings on impact**  Of the four studies that assessed real-time alerts to physicians at the time of medication ordering, three showed no effect, while one study suggested modest effectiveness. In contrast, all three of the studies incorporating asynchronous use of HIT systems showed improvements in drug monitoring adherence. The asynchronous alerts that employed a team of clinical pharmacists reported the greatest reductions in drug monitoring errors.  At a minimum, the limited studies to date suggest that passive (non-blocking) alerts are likely to have little or no effectiveness, and alerts that require physicians to navigate multiple steps seem likely to fail. | **Findings on moderators of effect**  Among the studies with real-time alerts the two that provided information to physicians without interrupting or blocking their work-flow were unsuccessful in improving laboratory testing rates. | **Further research indicated**  RCTs, multiple settings, patient outcomes and economic analyses |

| **Reference & topic** Hender 2000 ­– Impact of CDSS on practitioner performance and patient outcomes | | **Critical appraisal score**  11 |
| --- | --- | --- |
| **Studies and interventions**  10 SRs and RCTs – 1994 to 2000  No other details provided | **Limitations of studies**  Validity: design | **Limitations of review**  None noted |
| **Findings on impact**  None noted | **Findings on moderators of effect**  None noted | **Further research indicated**  None noted |

| **Reference & topic** Heselmans 2009 – Impact of CDSS on practitioner performance and patient outcomes in the ambulatory care setting | | **Critical appraisal score**  22 |
| --- | --- | --- |
| **Studies and interventions**  27 cluster RCTs, CCTs, CBA sand ITS – 1995 to 2008  Split between the US and Europe  All systems multi-dimensional, multi-step guidelines, provided prompts on screen, at the point of care with a majority for disease management | **Limitations of studies**  Validity: blinding, methodological reporting | **Limitations of review**  Quantity: computerised multi-dimensional guideline systems  Generalisability: specificity of organisational context  Heterogeneity: content, design, settings, metrics  Validity: retrieval bias, publication bias |
| **Findings on impact**  We found no evidence of an effect on patient outcomes, but the evidence is more mixed in terms of process of care. With success defined as at least 50% of the outcome variables being significant, none of the studies were successful in improving patient outcomes. Only 7 of 17 studies that investigated process outcomes showed improvements in process of care variables compared with the usual care group (comparison one). No incremental effect of the electronic implementation over the distribution of paper versions of the guideline was found, for either patient outcomes or the process outcomes (comparison two).  Outcomes were not consistent throughout all trials in earlier systematic reviews and patient outcomes were seldom studied, which is in line with the results of our review. However, conclusions were, in general, more positive than the main findings of our review, probably due to the exclusion of the more effective simple reminder systems and computerised paper-generated output and the differences in the definition of a successful intervention. | **Findings on effect moderators**  Reported reasons for failure to show an effect were work  overload and time pressure, low levels of usage, lack of integration within work-flow, technical problems, controversy over guideline, or highly complex suggestions  Ease, speed, and some control in the use of the system seemed to be critical success factors according the Discussion section of several included studies though further literature review to explore these topics was not done. | **Further research indicated**  High quality research design, patient outcomes  Benefits and harms, long-term effects, both direct and indirect costs |

| **Reference & topic** Hider 2002 – Impact of eRx/CDSS on practitioner performance and patient outcomes | | **Critical appraisal score**  21 |
| --- | --- | --- |
| **Studies and interventions**  52 SRs and RCTs – 1989 to 2000  Majority in US inpatient settings  Many from benchmark institutions | **Limitations of studies**  Validity: accounting for clustering, allocation concealment, baseline differences and adjustments, contamination, control of confounding, design, follow-up, metrics, power, selection bias, selection of controls | **Limitations of review**  Quantity: patient outcomes, primary care, RCTs  Heterogeneity:  Generalisability: home-grown systems, specialist settings  Validity: publication bias, retrieval bias, objectivity  Scope: inclusion of important outcomes and processes in evaluation |
| **Findings on impact**  A significant number of studies (44) have considered the effectiveness of CDSS in relation to prescribing. Most studies have reported that CDSS can improve practitioner performance especially at prescribing potentially toxic drugs (Level 1 evidence). Relatively few studies have considered patient health outcomes and several have failed to identify any additional patient benefits compared to physician-based care associated with CDSS (Level 1 evidence). The inability of some studies to find any improvement in health outcomes may be due to their lack of statistical power. The results from a meta-analysis of studies that considered electronic dose adjustment suggest that CDSS can reduce the frequency of adverse reactions and decrease length of hospital stay (Level 1 evidence).  CPOE effectively changed prescribing habits (Level III-3 evidence). Medication errors were reduced by the intervention but any beneficial effect on ADEs has not yet been established (Level III-3 evidence). CPOE increased the time required by physicians to order medication (Level III-3 evidence) but there is inconsistent evidence about whether consultations were lengthened (Level II evidence). CPOE was able to reduce patient charges even though their length of stay was not changed (Level II evidence). Sometimes physicians were not satisfied with the introduction of CPOE (Level II evidence). Corollary orders reduced medication errors but not patient length of stay or charges (Level II evidence). Most evaluations have been conducted in a small number of specialised settings (e.g. Harvard), where unique and sophisticated computer systems have been developed over more than a decade and where staff are generally well motivated to use these systems. Most studies of CPOE have also been located in hospitals. | **Findings on moderators of effect**  Key characteristics of situations where electronic prescribing systems have worked well include: organisations where there has been significant collaboration and leadership from senior clinicians and management, the use of fast, reliable systems that are uniform throughout the organisation and interface well with their operators, easy and direct access to machines (fixed or mobile) that are available where the clinical work is undertaken, and the provision of adequate resources including staff training and IT support. | **Further research indicated**  HFs & Technical specification    Economic evaluations of fully integrated systems, defining  costs and health outcomes, that explore the marginal costs and effectiveness of decision support  Determine whether decision support enables other professional groups to effectively and safely  assume roles  Compare the use of warnings at the time of prescribing versus the provision of alerts after the prescription has been completed  Explore the use of critiquing systems that check for alerts after prescriptions have been completed for audit and quality improvement purposes |

| **Reference & topic** Irani 2009 – Impact of EHR on patient satisfaction in the office-based outpatient setting | | **Critical appraisal score**  18 |
| --- | --- | --- |
| **Studies and interventions**  7 cross-sectional and pre/post-test studies – 2000 to 2007  All single site studies in US  Both commercial and home-grown systems | **Limitations of studies**  Validity: blinding, bias, control of confounding, design, methodological reporting, power, randomisation process  Scope: inclusion of relevant processes/outcomes in evaluation | **Limitations of review**  Heterogeneity: design, metrics, participants  Validity: metrics, bias |
| **Findings on impact**  The three cross-sectional studies examined found either neutral or positive patient attitudes about physicians’ EHR use during the outpatient visit, although these attitudes sometimes varied with the physician’s level of experience. Two of the four studies that evaluated patient satisfaction before and after EHR implementation found no effect on patient satisfaction results, whereas one found a positive result and another found a negative effect.  Three pre/post-test design studies gave percentage responses that can be quantitatively pooled. Using random effects modeling for the difference in rates yielded a pooled mean difference of 3.7% (95%CI 2.9% to 5.2%). This finding implies that patients’ satisfaction responses may be anywhere from approximately 3% to 5% higher after the EHR is introduced. | **Findings on moderators of effect**  Despite these limitations, these studies still yielded several useful and intriguing findings. For example, patient satisfaction was dependent on physician users’ experience with computers and their years in practice in one study. Another study similarly stated that patient satisfaction was higher with faculty physicians compared with resident physicians. Two other studies included more precise descriptions of physician concerns about EHR implementation: increased time needed to enter orders, increased time needed to provide complete documentation, and effect on rapport between physicians and patients. | **Further research indicated**  More rigorous studies on satisfaction needed |

| **Reference & topic** Jamal 2009 – Impact of HIT systems on practitioner performance and patient outcomes | | **Critical appraisal score**  16 |
| --- | --- | --- |
| **Studies and interventions**  23 SRs, RCTs, TSs and balanced block design studies – 1998 to 2006  Majority in US  Variety of settings and systems | **Limitations of studies**  Validity: allocation concealment, appropriateness of analysis, consistency of allocation, control of confounding, design, methodological reporting  Utility: sufficient details on intervention and context provided | **Limitations of review**  Quantity:  Heterogeneity: statistical vs clinical significance,  Validity: retrieval bias, publication bias |
| **Findings on impact**  Guided medication dosing appears to result in improved dose and frequency of choice. In the current review, a significant increase in physician compliance to recommended drug type and dosage were seen in all studies that measured provider outcomes, with improvement from 12% to 66%. Significant reduction of drugs ordered outside the recommended dosing range was also reported in two studies. All studies reported positive effects of systems on patient outcomes.  In the current review, 14 out of 17 studies that assessed the impact of HIT systems on practitioner performance, revealed a positive improvement in relation to their compliance with evidence-based guidelines. The impact of HIT systems on patient outcomes however inconsistent as only a small proportion of studies found benefits. For instance, only three studies shown positive improvement and the other five studies revealed either no change or adverse outcomes.  Nevertheless, the impact of HIT systems on clinical practices demonstrated consistency with other reviews. As has been shown in this review, wide-scale use of HIT systems, especially decision support and alerting systems, can increase the clinician’s adherence to clinical guidelines. | **Findings on moderators of effect** | **Further research indicated**  Standardised reporting for health informatics evaluations  Economic evaluation, patient outcomes  STIs |

| **Reference & topic** Jerant 2000 – Impact of EHR on practitioner performance and patient outcomes in the primary care setting | | **Critical appraisal score**  16 |
| --- | --- | --- |
| **Studies and interventions**  16 prospective studies with controls – 1978 to 1995  Many from benchmark institutions  All systems multi-functional | **Limitations of studies**  Validity: control of confounding, methodological reporting, randomisation process  Utility: sufficient details on intervention and context provided | **Limitations of review**  Generalisability: older systems, non-community settings |
| **Findings on impact**  Most studies evaluated the impact of EHR-generated reminders on provider compliance with health maintenance interventions. Findings were equally positive for both complete and hybrid EHRs with all but one trial reported positive results.  The use of either hybrid or complete EHRs in the primary care setting can be cautiously supported on the basis of their ability to improve provider compliance with screening measures, as well as to improve prophylactic and active problem treatment rates. | **Findings on moderators of effect**  When physicians are relied on to make reminders to patients, success appears more likely if they are supplied with patient-specific, printed or on screen point of care prompts rather than delayed feedback letters that are not linked to an encounter. It also appears that the ability of EHR-based reminder systems to increase the rates of screening manoeuvres is greater for those measures that can be quickly completed (e.g. serum cholesterol level) than for those that require a second appointment and are more inconvenient (e.g. Papanicolaou test). | **Further research indicated**  Newer systems, patient outcomes, better metrics, cost-effectiveness  Comparative trials of complete EHRs with hybrids  Technical specification effects |

| **Reference & topic** Kaushal 2003 – Impact of eRx /CDSS on medication safety | | **Critical appraisal score**  16 |
| --- | --- | --- |
| **Studies and interventions**  12 RCT, CTs and BAs – 1986 to 2001  Most in US inpatient settings from two benchmark institutions | **Limitations of studies**  Validity: power | **Limitations of review**  Generalisability: home-grown systems |
| **Findings on impact**  Of the five trials assessing eRx with CDSS, two demonstrated marked decrease in the serious ME rate, one an improvement in corollary orders, one an improvement five prescribing behaviours, and one an improvement in nephrotoxic drug dose and frequency. Of the seven trials assessing stand-alone CDSS, three demonstrated statistically significant improvements in antibiotic-associated MEs or ADEs, and one an improvement in theophylline-associated MEs. The remaining three had non-significant results.  These studies provide evidence that eRx with CDSS significantly decreases MEs and the rate of serious MEs at two institutions with home-grown systems. | **Findings on moderators of effect** | **Further research indicated**  Larger studies, assessing safety and clinical relevance, technical specification effects  Barriers to adoption, implementation in community hospital settings  Tools to assess commercial systems impact on medication safety |

| **Reference & topic** Mador 2009 – Impact of EHR on organisational efficiency in the critical care setting | | **Critical appraisal score**  20 |
| --- | --- | --- |
| **Studies and interventions**  12 MAs, RCTs, CTs, CBAs and ITSs – 1989 to 2008  Majority of systems interfaced with a HIS | **Limitations of studies**  Validity: control for confounding, Hawthorne effect  Scope: inclusion of relevant processes/outcomes in evaluation  Utility: sufficient details on intervention and context provided | **Limitations of review**  Quantity: EHR in critical care  Validity: publication bias |
| **Findings on impact**  Overall, three studies (25%) reported that time spent on documentation increased while five (42%) noted that there was no significant difference and four (33%) found that time spent on documentation decreased. The impact that the EHR had on time spent on direct patient care was similarly unclear. Of the 10 studies that measured this variable, five (50%) found that there was an increase in time available for direct patient care activities, while four (40%) studies found that there was no significant difference and one (10%) study found that there was a decrease in time available for these activities.  Due to the discrepant findings and several key methodological issues, the impact of an EHR in critical care on time spent charting and in direct patient care remains unclear. | **Findings on moderators of effect**  Few of the studies, which were pre/post-test designs, noted the point at which they were collecting their data in relation to “go-live” or the implementation date of the EHR. However, of the four studies that did report this information, data were collected at five months, 10 months, one year and two years post-implementation, respectively. Two authors reported that there was no significant difference in the time spent on documenting, while one found that there was a decrease. Interestingly, one study that made observations two years post-implementation found that nurses spent more time documenting compared to before the EHR was implemented. However, it was not possible to calculate the relationship between the length of time post-implementation and the amount of time spent charting or on direct patient care because of a lack of detail of the actual changes reported. | **Further research indicated**  Standardised metrics and reporting for work tasks  Multi-disciplinary methods  Hawthorne effect minimised  Time and motion analysis preferable, entire unit’s work-flow, longitudinal data collection  More detailed reporting on intervention and context |

| **Reference & topic** Mitchell 2000 – Impact of HIT systems on organisational efficiency, practitioner performance, patient outcomes and satisfaction in the primary care setting | | **Critical appraisal score**  17 |
| --- | --- | --- |
| **Studies and interventions**  89 studies of variable design – 1983 to 1997 | **Limitations of studies**  Validity: unit of analysis | **Limitations of review**  Quantity: primary care, patient outcomes |
| **Findings on impact**  Most of the 89 studies in this review found positive effects of decision support for improvements in immunisations and preventive care and reductions in prescribing costs and unnecessary tests. Computer use during consultations lengthened the consultation. Reminder systems for preventive tasks and disease management improved process rates, although some returned to pre­intervention levels when reminders were stopped. Use of computers for issuing prescriptions increased prescribing of generic drugs, and use of computers for test ordering led to cost-savings and fewer unnecessary tests. There were no negative effects on those patient outcomes evaluated.  The most fruitful areas of current research are preventive care, prescribing support, chronic disease monitoring, test ordering, and hospital referral. | **Findings on moderators of effect**  Five themes emerged that could prove major barriers to successful implementation of computers; privacy, the patient-provider relationship, costs, time and training. | **Further research indicated**  Integration of computer into consultation  Impact on nursing and other primary care team members |

| **Reference & topic** Montgomery 1998 – Impact of CDSS for hypertension on practitioner performance and patient outcomes | | **Critical appraisal score**  14 |
| --- | --- | --- |
| **Studies and interventions**  7 RCTs – 1976 to 1990  Majority in North America | **Limitations of studies**  Validity: blinding, follow-up, methodological reporting, metrics, randomisation process, unit of analysis | **Limitations of review**  Quantity: RCTs  Heterogeneity: intervention targets, metrics  Generalisability: non-sophisticated systems  Validity: control for confounding |
| **Findings on impact**  From these results, it is still not certain whether computers have a favourable effect on the management of hypertension. The three outcome dimensions measured were patient administration, practitioner performance, and BP control, with six of seven trials examining more than one outcome. Patient uptake/administration was evaluated in five trials, four of which reported significant improvement using a computer. Practitioner performance was evaluated in three trials, two of which reported improvement using a computer. Control of BP was evaluated in six trials, two of which reported improvement using a computer.  Two previous reviews that examined the effects of computers on patient care were more positive in their findings. Both these reviews awarded higher trial quality scores to trials that randomised by practice, but neither review seemed to take account of possible exaggeration of results when outcomes were analysed by patient.  This review shows that computers may have a favourable influence on the uptake and administration of patients in hypertension management. Computer use in this area should be encouraged, particularly with regard to case-finding and follow-up. The results presented here do not seem to support any benefit in using computers in terms of practitioner performance and BP control in patients. | **Findings on moderators of effect** | **Further research indicated**  Newer technology |

| **Reference & topic** Niazkhani 2009 – Impact of CPOE on organisational efficiency in the inpatient setting | | **Critical appraisal score**  22 |
| --- | --- | --- |
| **Studies and interventions**  45 studies of variable design – 1993 to 2007  Most in adult inpatient settings with academic affiliation  Most of commercial systems | **Limitations of studies**  Scope: inclusion of relevant processes/outcomes in evaluation  Utility: sufficient details on intervention and context provided | **Limitations of review**  Heterogeneity: systems, settings  Generalisability: commercial systems  Validity: retrieval bias, inclusion/exclusion criteria, sub-groups |
| **Findings on impact**  Our review shows that the impact of CPOE on clinical work-flow is double-edged. On the one hand, it shows that the implementation of CPOE has resolved many disadvantages associated with the work-flow in paper-based practices. Many CPOEs have improved work-flow efficiency in terms of the legibility and completeness of orders; the availability of decision support features and order sets; the remote accessibility of the system; the possibility to view the same patient data simultaneously by multiple providers; and fewer work interruptions due to asynchronous communication. They have also decreased verbal orders and improved order countersignature. Furthermore, these systems contributed in time efficiency in term of shorter turnaround times. On the other hand, our review also reveals that the implementation is accompanied by difficulties in work-flow, mainly due to changes in the structure of pre-implementation work. Negative effects included time-consuming user-system interaction; the removal of visual clues available in paper-based systems; the enforcing of predefined and stepwise order of activities as well as role-based relationship between providers; emerging problems in the synchronisation of interdependent tasks; and the restricting of opportunities for team-wide discussions. | **Findings on moderators of effect** | **Further research indicated**  Multi-disciplinary methods  Macro effect, especially on collaborative work-flow  Control of confounding, across multiple sites or settings |

| **Reference & topic** Oren 2003 – Impact of eRx/CDSS on medication safety | | **Critical appraisal score**  14 |
| --- | --- | --- |
| **Studies and interventions**  11 controlled studies – 1993 to 2001  Most from two benchmark institutions | **Limitations of studies**  None noted | **Limitations of review**  Quantity: controlled studies, patient outcomes  Generalisability: home-grown systems, selected sites |
| **Findings on impact**  Only three studies evaluated the impact of CPOE on MEs and ADEs. One study observed a reduction in both preventable and potential ADEs when a CPOE system was implemented. The rates of non-intercepted serious MEs (errors that are not intercepted before injury) and non-intercepted potential ADEs (errors that by chance resulted in no injury) were significantly reduced with CPOE. Furthermore, these reductions were observed across all levels of severity for non-intercepted serious MEs. In another trial evaluating ME and ADEs, decreases in MEs were observed with CPOE for all major categories of MEs and were further categorised as “missed dose” and “non-missed dose.” The non-missed dose ME rate per 1000 patient-days decreased by 81%. The missed dose error rate per 1000 patient-days climbed significantly with the use of CPOE. The authors suggested that each missed dose error resulted in 15 minutes of extra work for nursing and pharmacy personnel. The non-intercepted serious ME rate per 1000 patient-days also fell significantly.  Our results confirm our belief that very few controlled studies have evaluated the impact of CPOE on patient outcomes. | **Findings on moderators of effect**  The authors of the study where the missed dose error rate increased post-CPOE indicated that changes in patient acuity, pharmacy staffing, and other work-flow changes may have contributed. Additionally, only one study was identified that evaluated both our aims. The study assessed a CDSS for disease management. The number of ordered anti-infective agents decreased, the duration of therapy was reduced, the cost of anti-infectives decreased, and the length of hospital stay was reduced. ADEs caused by anti-infectives decreased compared with the pre-intervention period. In this system, the physician could over-ride and did so approximately half the time. When over-ridden, the mean number of prescribed agents increased, the duration of therapy increased, the mean number of doses increased, the mean cost of agents increased, and the mean length of stay climbed.  Even fewer have assessed the appropriateness of use of CPOE with very little or no evidence on the appropriateness of the use of each technology was found. | **Further research indicated**  Efficacy versus effectiveness  Appropriateness of use |

| **Reference & topic** Pearson 2009 – Impact of eRx/CDSS on practitioner performance | | **Critical appraisal score**  23 |
| --- | --- | --- |
| **Studies and interventions**  56 RCTs, Quasi-experimental studies and ITSs – 1991 to 2007  Majority in North American ambulatory care settings  Majority active systems for initiating medication, the rest on monitoring | **Limitations of studies**  Validity: baseline differences and adjustments, power  Utility: sufficient details on intervention and context provided | **Limitations of review**  Heterogeneity: study designs, methods and settings, intervention targets and outcomes  Scope: consider all processes and outcomes relevant to finding  Validity: retrieval bias, publication bias, inclusion/exclusion criteria, summary measure/metrics |
| **Findings on impact**  Overall, 36 of 38 studies on initiating treatment demonstrated at least one positive prescribing outcome in favour of CDSSs. With success defined as at least 50% of the prescribing outcomes reported being significant, 19 were successful. Of the 26 studies providing support before drug selection 24 showed improvements in at least one outcome with 12 successful. Importantly, one study on preventive care found the CDSS was significantly inferior to its comparator on at least one outcome measure. All 12 studies providing support after drug selection reported improvements in at least one outcome with seven successful. Of the 23 studies on monitoring existing therapy, 18 demonstrated positive results on at least one outcome with eight successful.  CDSSs also appeared to be effective for monitoring therapy, particularly using laboratory test reminders with four of seven studies successful. None of the studies addressing stopping therapy demonstrated impacts in favour of CDSSs over comparators. The most consistently effective approaches used active systems to support the fine-tuning of existing therapy by making recommendations to improve patient safety, adjust the dose, duration or form of prescribed drugs, or increase the laboratory testing rates for patients on long-term therapy. Cardiovascular disease was the most studied clinical target but few studies were successful. | **Findings on moderators of effect**  Consistent with previous reviews, active decision support appeared to be more effective than passive. Our finding that multi-faceted interventions appeared no more effective than decision support alone conflicts with some reviews but not others. Further, consistent with previous reviews, CDSSs implemented in inpatient as opposed to ambulatory settings appeared to be more effective.  At the time of initiating therapy, CDSSs appear to be somewhat more effective after drug selection has occurred rather than before, (7/12 studies successful vs 12/26) respectively. | **Further research indicated**  Standardised reporting for health informatics evaluations  Benefits and risks assessed longitudinally, economic analyses, better metrics, e.g. appropriateness of prescribing  Decision support for stopping prescriptions  Technical specification effects  HFs and socio-technical issues on barriers/success |

| **Reference & topic** Poissant 2005 – Impact of EHR and CPOE on organisational efficiency | | **Critical appraisal score**  25 |
| --- | --- | --- |
| **Studies and interventions**  20 RCTs, post-test control studies, and BAs – 1993 to 2003  Variety of settings and systems studied | **Limitations of studies**  Scope: inclusion of relevant processes/outcomes in evaluation  Utility: sufficient details on intervention and context provided | **Limitations of review**  Heterogeneity: systems, decade evaluated  Validity: inclusion/exclusion criteria, sub-groups |
| **Findings on impact**  The use of bedside terminals and central station desktops saved nurses, respectively, 24.5% and 23.5% of their overall time spent documenting during a shift. Our results show that using bedside or point of care systems increases documentation time of physicians by 17.5%. In comparison, the use of central station desktops to document clinical notes is slightly less time-consuming, with a weighted mean of 8.2%. The use of central station desktops for CPOE was time inefficient in all three studies, consuming from 98.1% to 328.6% more time per working shift. The weighted mean relative time difference across these CPOE-oriented studies was an increase in documentation time of 238.4%. Several studies have shown that computers increase the completeness of information being documented. | **Findings on moderators of effect**  All studies examining the impact of EHR over working shift periods, reporting favorable time efficiencies compared to those with patients or patient encounters as the sampling units.  In Studies that conducted their evaluation process relatively soon after implementation of the EHR tended to demonstrate a reduction in documentation time in comparison to the increases observed with those that had a longer time period between implementation and evaluation.  Our results did not identify a clear trend toward enhanced time efficiency despite the increased speed of computers, the availability of customised software, and the large array of user-interfaces and input devices. | **Further research indicated**  HFs and socio-technical issues on barriers/success  Macro impact on organisational efficiency  Cost-benefit for various end-users  Standardised metrics and reporting for task times |

| **Reference & topic** Randell 2007 – Impact of CDSS in nursing on practitioner performance | | **Critical appraisal score**  17 |
| --- | --- | --- |
| **Studies and interventions**  8 RCTs, CTs, CBAs and ITS – 1991 to 2005  Majority in UK primary care setting  Anticoagulation and telephone triage exclusively | **Limitations of studies**  Validity: contamination, power  Scope: inclusion of relevant processes/outcomes in evaluation  Utility: sufficient details on intervention and context provided | **Limitations of review**  None discussed |
| **Findings on impact**  In summary, three studies compared nurses with decision support to nurses without. In one study CDSS use improved performance, while in another it was associated with poorer performance, and no study found an impact of CDSS on patient outcomes. Three RCTs comparing nurses using decision support with physicians for anticoagulation therapy found no significant difference in terms of patient outcomes, suggesting that decision support may help nurses to manage anticoagulation as effectively as physicians. The two studies of triage for first contact care suggest decision support to be beneficial in terms of performance, with significantly decreased GP work-load when nurses used decision support. While one study suggests that decision support is detrimental to patient outcomes, another study suggests that it is decision support to be beneficial in terms of some patient outcomes. Current evidence on the benefit of systems for telephone triage is equivocal and no clinical trials to date have evaluated their use in face-to-face consultations. | **Findings on moderators of effect**  For a number of studies in this review, the results suggest that the failure lies with the protocols on which the decision support is based. | **Further research indicated**  CDSS for nurse-led chronic disease management  Technical specification effects  HFs and barriers/success  MRC framework for complex interventions, separating out the effect of the protocol versus the guideline  Randomisation at practitioner, unit level  Multi-disciplinary methods |

| **Reference & topic** Reckmann 2009 – Impact of eRx/CDSS on medication safety in the inpatient setting | | **Critical appraisal score**  20 |
| --- | --- | --- |
| **Studies and interventions**  13 BAs, ITSs, cross-sectional, crossover and comparative cohort studies – 1998 to 2007  Split between US and Europe, many academically affiliated | **Limitations of studies**  Validity: design, detection bias  Utility: sufficient details on intervention and context provided | **Limitations of review**  Heterogeneity: metrics |
| **Findings on impact**  Evidence of the effectiveness of eRx to reduce PE is limited in quantity and further reduced by limitations of studies. Nine of the 12 studies (two studies reported the same data) demonstrated a significant decrease in PE rates ranging from 29 to 96%. Five of the 12 studies examined error severity, but only two clearly defined their severity categories. Most studies made reference to or reported some data to support the claim that eRx is effective at improving the completeness of orders and at reducing these more minor errors. However, limited conclusions can be drawn regarding changes in the severity of errors following the implementation of eRx. The question of whether CPOE is effective at reducing errors of greater severity remains unanswered.    New errors introduced by CPOE included the selection of an inappropriate dosage form for a required route (e.g. capsules for intravenous administration), selection of an inappropriate product, incorrect dose, frequency, or formulation from a dropdown menu, inappropriate use or selection of default doses and missed drug allergies. Increases in duplicate orders post-CPOE were reported in several studies. Increased frequency of failure to discontinue drugs no longer required and increased drug monitoring errors were also noted. | **Findings on moderators of effect**  One study reported a very high over-ride rate for drug allergies and high severity drug interactions. One study reported that the chemotherapy unit CPOE failed to include infusion solution diluents if a central venous line was not listed. | **Further research indicated**  Internally valid, powerful, commercial systems, across multiple sites  Standardised metrics and reporting of MEs  Development of a standardised nomenclature of CPOE-related errors  Technical specification effects  HFs for barriers/success |

| **Reference & topic** Rothschild 2006 – Impact CPOE on organisational efficiency, practitioner performance and medication safety in the inpatient setting | | **Critical appraisal score**  16 |
| --- | --- | --- |
| **Studies and interventions**  18 RCTs, BAs and TSs – 1993 to 2003  Majority in general adult inpatient settings with academic affiliation | **Limitations of studies**  Validity: design, power | **Limitations of review**  Quantity: RCTs, critical care  Heterogeneity: study designs, outcomes  Generalisability: home-grown systems, research settings |
| **Findings on impact**  The studies on prescribing provide the strongest evidence for the benefits of CPOE on patient outcomes. Surrogate outcome improvements associated with CPOE included reduced medication and/or overall hospital costs and increased selection of appropriate drugs and correct drug dosing. Inclusion of decision support during the ordering process not only reduces medical errors, but also increases the efficiency of healthcare delivery and the use of evidence-based clinical guidelines.  Studies of CPOE and diagnostic test ordering reveal that while display of charges for laboratory and radiologic test orders does not reduce the number of ordered or completed tests, reminders displayed with redundant orders successfully decrease redundant and unnecessary order requests.  Of the eight studies regarding injury prevention or prophylactic measures and non-medication-related resource utilisation, only a single study failed to demonstrate a beneficial effect as a result of CPOE. | **Findings on moderators of effect**  A study which failed to demonstrate a beneficial effect as a result of CPOE was able to improve guideline adherence with a modification to the delivery of the CDSS. | **Further research indicated**  Critical care, long-term effects, patient outcomes  RCTs sufficiently powered |

| **Reference & topic** Schedlbauer 2009 – Impact of eRx/CDSS on practitioner performance, patient outcomes and medication safety | | **Critical appraisal score**  22 |
| --- | --- | --- |
| **Studies and interventions**  20 RCTs, CBAs, ITSs and TSs – 1994 to 2007  Most in US inpatient settings | **Limitations of studies**  Validity: methodological reporting, internal validity | **Limitations of review**  Quantity: certain alerts (drug–drug interaction alerts, drug-disease contraindication alerts and dosing guidelines based on age)  Heterogeneity: providers, patients, settings, intervention, timing of evaluation, metrics  Validity: retrieval bias, sub-groups, title/abstract review/screening |
| **Findings on impact**  Of the 27 alerts, 23 resulted in statistically significant improved prescribing behavior and/or reductions in MEs. Most of the changes noted were clinically significant. Three of the remaining four showed statistically non-significant improvements in prescribing. Of the four alert types examined with respect to patient outcomes, three had a positive and statistically significant impact *Drug allergy warnings, default dosing, drug*-*drug interaction warnings.* Most studies that evaluated more advanced alert types with statistically significant effects shown in 21 out of 23.  We conclude that most empiric studies evaluating the effects  of computerised prompts and alerts on prescribing behavior showed positive, and often substantial, effects. | **Findings on moderators of effect**  Three of the alert types with lacking benefit showed weaknesses in their methodology or design.  No empiric studies assessing the effectiveness of different approaches to the design and display of alerts and prompts were found. | **Further research indicated**  Powerful, internally valid, alerts for drug–drug interaction, drug-disease contraindication alerts and dosing guidelines based on age, patient outcomes, health service management outcomes  Standardised metrics and reporting of MEs and other sup-optimal prescribing  HFs and barriers/success |

| **Reference & topic** Shachak 2009 Impact of EHR on consultation and satisfaction | | **Critical appraisal score**  15 |
| --- | --- | --- |
| **Studies and interventions**  14 studies of variable design – 1997 to 2007  Most in primary care settings | **Limitations of studies**  None noted | **Limitations of review**  Quantity: EHR and PDC  Generalisability: isolated findings |
| **Findings on impact**  This review indicates that while having a positive impact on information-related tasks and information exchange (the first function of the medical interview), particularly about medications, EHR had a negative impact on the second function – psychological and emotional talk, establishing rapport with patients and patient centredness. There is some indication that the introduction of the EHR organises encounters around data gathering demands rather than patients’ narratives although in some cases the introduction of computers added complexity to the visit and introduced new tasks. Our analysis suggests that physicians rarely used the computer for the third function of the medical interview, patient education and behavioural management. Patients were usually satisfied with their physician’s use of EHR. | **Findings on moderators of effect**  Several factors affected PDC in computerised setting, including  cognitive limitations, physician characteristic and spatial organisation of the clinic. It has been suggested that both computer use and communicating with the patient require the physician’s focused attention, and that multi-tasking or computer use in the background are not possible. Physicians’ characteristics affected their ability to handle the additional cognitive load imposed by computer. Computer mastery enhanced PDC in a computerised setting. Typing skills were viewed by physicians as crucial for using the EHR effectively during the clinical encounter, reduced physicians’ need to focus attention on the computer, and positively affected PDC. Physicians’ ability to navigate the computer, search for and organise information was also associated with their ability to communicate with patients effectively. Physicians’ experience and baseline communication skills have been proposed to affect PDC in a computerised setting. Patients who were seeing residents were significantly more likely to agree that the EHR had a negative effect on the time physicians spent talking, looking at and examining them than patients seeing faculty physicians. Similar trends, though not statistically significant, were reported by the physicians. Basic communication skills were highly related to the quality of PDC, and the computer seemed to amplify both positive and negative pre-implementation communication patterns. Another physician characteristic which influenced PDC was their behavioural style. This issue is discussed in detail below. Spatial organisation of the computerised environment affected physicians’ ability to utilise the EHR effectively. In some cases location of the screen interfered with eye contact. Fixed positioning limited physicians’ ability to face patients directly, sometimes forcing them to shift their body or move the chair to face patients. In other cases, location of the screen or using flat monitors on mobile arms helped sharing information with patients; thereby facilitating PDC and patient education. However, in another study, post-shift surveys of emergency physicians did not find significant difference in perceived effect on PDC between using desktop and mobile computers. In one study, the accessibility of computer created a need to immediately enter data into the EHR resulting in a conflict between this need and the need to pay full attention to the patient. On the other hand, the opportunity to access EHR from different locations gave both patients and physicians a feeling of seamless communication over time and location. | **Further research indicated**  Cognitive elements in using an EHR during the patient-provider encounter, such as focus of attention, memory load and automaticity of actions  Methodologies drawn from Cognitive Sciences and Human–Computer Interaction (e.g. Cognitive Task Analysis)    Best practices of EHR use and effective strategies for incorporating it into PDC  When and how to introduce the EHR to students and residents |

| **Reference & topic** Shamliyan 2008 – Impact of eRx w/o CDSS on medication safety | | **Critical appraisal score**  13 |
| --- | --- | --- |
| **Studies and interventions**  12 RCTs, BAs and controlled observational studies – 1990 to 2005  Many from benchmark institutions | **Limitations of studies**  Validity: detection bias | **Limitations of review**  Generalisability: academic affiliates  Heterogeneity: significant unexplained by design or system  Validity: publication bias, metrics, control for confounding |
| **Findings on impact**  Compared with handwritten orders, 80% of studies (8 of 10 studies) reported a significant reduction in total MEs, 43% in dosing errors, and 37.5% in ADEs. The use of computerised orders was associated with a 66% reduction in total MEs in adults (OR 50.34, 95%CI 0.22 to 0.52) and a positive tendency in children (p=.03).  Total adverse events were lessened after CPOE in three studies with a positive but not statistically significant tendency to reduce ADEs in four studies. The use of CPOE would prevent 9 ADEs per 1,000 prescriptions in paediatric and 12 ADEs per 1,000 prescriptions in an adult population (Evidence level I) and several uncontrolled interventions and observational studies (Evidence levels II2–II3) confirmed previous contentions that implementation of CPOE was associated with a significant reduction in MEs in adult and paediatric populations.  The use of CPOE was not associated with a substantial improvement in patient safety. Existing evidence (levels I–II3) suggests a significant reduction in MEs is associated with implementing CPOE, but the effects are not consistent among patient populations and clinical settings and are compromised by the design of studies. Sites that use CPOE still experience high rates of MEs and ADEs. | **Findings on moderators of effect**  We could not quantify the functionality of the CPOE software used and assumed that any differences would not modify the impact on outcomes.  The test for heterogeneity was significant (p<.001) and could not be explained with meta-regression: neither year of publication (p=.9), the presence of control group (p=.8), the target population (p=.6), nor clinical setting (p=.9) affected the association between CPOE and ADE. Unexplainable significant heterogeneity in the results compromised pooled relative risks.  The test for heterogeneity was significant (p<.001). The benefit of computerised orders was larger when the ME rate was more than 12% with handwritten orders (p=.02). A greater decrease in dose errors was observed in studies with a control group compared with pre/post-test CPOE analyses (p<.001). Randomised intervention demonstrated a 72% reduction in risk of wrong dose MEs using CPOE (OR 50.28, 95%CI 0.15 to 0.52) with 19 avoided MEs per 1,000 orders.  The risk of harmful events related to MEs was three-fold in children compared with adults with the same rate of MEs, although the effects of CPOE were less potent in paediatric settings. The use of CPOE eliminated MEs in specialised units that prescribed fewer groups of drugs, and therefore had a higher probability of positive effects from the interventions. | **Further research indicated**  Standardised metrics and reporting for MEs  CPOE with combined strategies |

| **Reference & topic** Shebl 2007 – Impact of eRx/CDSS for antibiotics on practitioner performance and patient outcomes | | **Critical appraisal score**  17 |
| --- | --- | --- |
| **Studies and interventions**  10 RCTs and CBAs – 1989 to 2006  Most in US  Many from benchmark institutions | **Limitations of studies**  Validity: baseline differences and adjustments, contamination, control for confounding, methodological reporting, power, selection of control,  Scope: inclusion of relevant processes/outcomes in evaluation  Utility: sufficient details on intervention and context provided | **Limitations of review**  Heterogeneity: methods, metrics, systems  Generalisability: limited sites  Validity: retrieval bias, inclusion/exclusion criteria |
| **Findings on impact**  Eight of the ten studies identified a statistically significant advantage for decision support. The positive results associated with decision support in this review are in line with the conclusion of several large systematic reviews evaluating the use and benefit of decision support in general.  The results of this review indicate that decision support may be a useful tool to help optimise antibiotic use and improve patient care. | **Findings on moderators of effect** | **Further research indicated**  Standardised reporting for health informatics evaluations  Across different settings  Multi-disciplinary methods  Socio-technical issues on barriers/success |

| **Reference & topic** Shekelle 2006 – Costs and benefits of HIT systems | | **Critical appraisal score**  20 |
| --- | --- | --- |
| **Studies and interventions**  256 SRs, MAs, and hypothesis-testing and predictive analyses  Majority in US outpatient settings with academic affiliation  Many from benchmark institutions | **Limitations of studies**  Validity: contamination, control for confounding, design, bias  Scope: inclusion of relevant processes/outcomes in evaluation  Utility: sufficient details on intervention and context provided | **Limitations of review**  Quantity: economic analyses, multi-functional commercial EHRs  Generalisability: home-grown systems, academic affiliates, costs  Validity: retrieval bias, publication bias |
| **Findings on impact**  Limited empiric evidence exists to support a benefit for HIT systems use in paediatrics in the areas of medication safety, clinical decision support, process improvement, and cost reduction.  A small set of high quality studies shows that implementation of a comprehensive ambulatory EHR improves quality of care. Available evidence focuses primarily on the impact of ambulatory EHRs on decreasing overused health services by enhancing access to data, providing capabilities for real-time analysis of clinical data, and acting as platforms for decision support. Ambulatory EHRs improve the structure of care delivery, improve clinical processes, and enhance outcomes. Most available evidence shows the effects of ambulatory EHRs on processes of care.  The main quantifiable benefits of an EHR were savings from data capture and access; decision support to improve efficiency, quality, and safety of care; business management related to staffing, billing, and overheads; and streamlining patient flow. All the cost-benefit analyses of an EHR predicted that the financial benefits would significantly outweigh the costs, in a timeframe that varied from three to thirteen years, for large organisations and multi-functional EHRs. There is some evidence regarding the positive economic value of implementing component parts of an EHR, with models suggesting that many of the benefits do not accrue unless a broadly functional system is implemented. | **Findings on moderators of effect**  Studies identified a large number of barriers to the implementation of HIT systems. These barriers can be classified as situational barriers (including time and financial concerns), cognitive and/or physical barriers (including users’ physical disabilities and insufficient computer skills), liability barriers (including confidentiality concerns), and knowledge and attitudinal barriers. | **Further research indicated**  Internally valid, within community, across multiple settings, day-to-day use, commercial systems  Standardised reporting for health informatics evaluations  Development of methods and instruments directed at evaluation of externally developed systems  Functionality specific effects  Paediatric specific research, work-flow, cost-benefit, medication safety across multiple settings  Simulation modeling on cost-benefit to supplement hypothesis testing  Simulation modeling on flow of costs and benefits across multiple perspectives |

| **Reference & topic** Shekelle 2009 – Costs and benefits of HIT systems | | **Critical appraisal score**  21 |
| --- | --- | --- |
| **Studies and interventions**  156 studies of variable design  Split between outpatient and inpatient settings  Many from benchmark institutions | **Limitations of studies**  Validity: assumptions  Scope: inclusion of relevant processes/outcomes in evaluation  Utility: sufficient details on intervention and context provided | **Limitations of review**  Generalisability: economic analyses,  Validity: retrieval bias, publication bias, inclusion/exclusion criteria |
| **Findings on impact**  New studies from benchmark institutions demonstrate both the potential and the limitations of improvements in care that may be realistically achieved in the near future with broader implementation of multi-functional EHRs. Improvements in the processes of care and reductions in the number of preventable ADRs were reported in some, but not all, studies. Even with the use of HIT systems the standards of some processes of care remain far below what is desired (often less than half of eligible patients received recommended care despite the intervention). Furthermore, problems abound with physicians ignoring or over-riding recommended care.    Although still rare in number, there are more published studies of commercial systems of which we found three kinds. The first assessed the effect of adding new functionalities to existing systems. These study results were similar to those reported by field leaders: most demonstrated modest benefits, some found no benefits and a small number showed marked benefits. This supports the contention that the findings of studies from the field leaders about the effect of adding functionalities to existing EHRs is probably generalisable to other institutions. The second type of study, of which there was only one, assessed the effects of the implementation of a HIT system on broader organisational measures. The authors, contrary to their expectations, found that implementing an EHR resulted in perceptions of a more hierarchical organisation. This supports the hypothesis that an organisation’s culture and HIT systems implementation interact in complex ways. The last kind of study we found was an assessment of the effect of introducing a multi-functional commercial EHR into a healthcare setting where none had existed previously. This type of study is perhaps the most important for organisations considering purchasing an EHR, and we found only two.  Cost and cost-effectiveness data are still limited. Our prior review concluded that five cost-benefit studies consistently predict that implementation of an EHR can be financially viable for individual organisations or through a nationwide implementation with high levels of healthcare information exchange and interoperability. However, there are important caveats:  • all the studies are predictive analyses that are based on many analytical assumptions and limited empirical data  • the strength of the evidence is weak  • all the studies assumed that the EHR had multiple functionalities that include, at a minimum, health information and data storage, administrative processes, CDSS and results  management, as well as information exchange capabilities  • the functional capability of an EHR is critical to the benefits accrued  • both the cost and the benefit of attaining interoperability among EHRs are directly proportional to the level of data exchange achieved.  Unfortunately, the updated review did not provide significant additional information regarding the costs and benefits of fully functional EHRs. Analyses of the costs and benefits of adding CPOE indicate that the results may be different depending on context. | **Findings on moderators of effect**  The primary barriers to adoption were cost and perceived resistance on the part of physicians. Recently, a case study of implementation was published that looked at the factors behind a successful implementation of an EHR, and compared them to expectations from Rogers’ Theory of the Diffusion of Innovations and to prior literature. These factors included:  • consultation before implementation  • consensus about the need for a system and which one was best  • prioritisation and “drive” from the management team  • competent IT project leader and team  • tested, user-friendly and intuitive system that could be used with little training  • potential for development of the system  • medication order entry | **Further research indicated**  Standardised reporting for health informatics evaluations  Usability studies |

| **Reference & topic** Shiffman 1999 – Impact of CDSS on practitioner performance, patient outcomes and satisfaction | | **Critical appraisal score**  14 |
| --- | --- | --- |
| **Studies and interventions**  25 RCT, CT and TS – 1992 to 1998  Most in US, a variety of settings included  Many from benchmark institutions  Computerised and computer-generated prompts, most prompted documentation, all provided patient-specific recommendations | **Limitations of studies**  Validity: design  Scope: inclusion of relevant processes/outcomes in evaluation  Utility: sufficient details on intervention and context provided | **Limitations of review**  Heterogeneity: study designs and settings, systems, implementation strategy  Validity: publication bias, critical appraisal |
| **Findings on impact**  Four studies looked at documentation and found improvement in each case. In 14 of 18 studies evaluating guideline adherence some level of improved adherence was described. In several reports, improved adherence occurred for some of the measured outcomes but not for all. Few studies examined patient outcomes to validate the effectiveness of the systems. | **Findings on moderators of effect**  Although systems delivered patient-specific recommendations – in most cases the advice was made available concurrently – providing recommendations in this manner was neither necessary nor sufficient to ensure adherence. Failure to improve adherence using computer-based strategies was reported in four studies. An attempt to improve preventive care guideline adherence for hospitalised patients failed because of functional and systemic barriers that interfered with providing preventive care to inpatients. One study of prevention and management of pressure ulcers was unable to show any effect of the computer-based intervention o nursing decision making. In that case, the authors concluded that there was not enough gain for the effort of data entry. A system designed to influence decision making in emergency room patients with back pain failed because of general confusion regarding the utility of plain x-rays in these patients and the fact that recommendations were not enforced. Finally, in a study of diabetes management guidelines, compliance improved to the same degree in both control and intervention groups; the authors questioned study design issues.  In both studies with negative evaluations of user satisfaction arduous data entry was suggested as a reason for poor system acceptance. | **Further research indicated**  None noted explicitly |

| **Reference & topic** Shojania 2009 – Impact of CDSS on practitioner performance | | **Critical appraisal score**  22 |
| --- | --- | --- |
| **Studies and interventions**  28 RCTs and quasi-RCTs – 1993 to 2008  Majority in US outpatient settings, all studies in inpatient settings from two benchmark institutions  Point of care prompts only | **Limitations of studies**  Validity: allocation concealment, baseline differences and adjustment, follow-up, methodological reporting, power, unit of analysis  Utility: sufficient details on intervention and context provided | **Limitations of review**  Heterogeneity: systems  Validity: summary measure/metrics, sub-groups, multiple comparisons |
| **Findings on impact**  Across 32 comparisons, computer reminders achieved small to modest improvements in care. Using the post-intervention difference between study groups, the median improvements in process adherence associated with computer reminders were: 4.2% (IQR, 0.8% to 18.8%) across all process outcomes, 3.3% (IQR, 0.5% to 10.6%) for improvements in prescribing behaviours, 3.8% (IQR, 0.5% to 6.6%) for improvements in vaccination, and 3.8% (IQR, 0.4% to 16.3%) for test ordering behaviours. The absolute improvement in process adherence was less than 4% for half of the included comparisons. Even when we included the best outcome from each comparison, the median improvement was only 5.6%. With the upper quartile of reported improvements beginning at a 15% increase in process adherence, some studies clearly did show larger effects. However, we were unable to identify any study or reminder features that predicted larger effect sizes, except for a statistically significant (albeit unadjusted for multiple comparisons) difference in effects seen in studies involving the CPOE at BWH. | **Findings on effect moderators**  We were unable to identify any study or reminder features that predicted larger effect sizes, except for a statistically significant (albeit unadjusted for multiple comparisons) difference in effects seen in studies involving the CPOE at BWH. A trend towards larger effects was seen for reminders that required users to enter a response in order to proceed, but this finding may have been confounded by the uneven distribution of studies from BWH. The finding that comparisons of computer reminders alone versus usual care reported larger effect sizes than comparisons involving computer reminders and other co-interventions represented an unexpected finding. | **Further research indicated**  Technical specifications and HFs for reliable improvement |

| **Reference & topic** Sintchenko 2007 – Impact of CDSS on practitioner performance and patient outcomes | | **Critical appraisal score**  17 |
| --- | --- | --- |
| **Studies and interventions**    24 RCTs – 1990 to 2003  Split between US and Europe, majority in primary care settings  Majority of systems provided support for prescribing for chronic diseases | **Limitations of studies**  Validity: methodological reporting | **Limitations of review**  Quantity: RCTs, patient outcomes  Heterogeneity: study setting, systems, clinical problems, clinician and patient selection, methods of intervention, metrics  Validity: retrieval bias, publication bias |
| **Findings on impact**  Overall, 13 (54%) of the studies showed a positive result, and 11 (46%) were negative in Level 1 and 2 outcomes. Of the positive studies, only one showed an improvement in Level 1 patient outcomes with a majority of studies (50% or 12/24) demonstrating an improvement only in surrogate outcomes and other variables (Level 2 outcomes) as indirect measures of patient outcomes (p=0.04). Critiquing and consultative systems demonstrated positive impact in 83% and 50% of controlled studies, respectively. Furthermore, only one consultative system which supported drug dosing as a part of CPOE showed an improvement in patient length of stay (Level 1 outcome) along with a decrease of inappropriate dosing and frequency (Level 2 outcomes). All systems targeting clinical decisions related to acute disease or acute exacerbation of chronic disease improved patient outcomes compared with 38% of systems focused on the management and treatment of chronic conditions (p=0.005). For example, no benefit with respect to the management of asthma, angina, or major depression was observed. Two of five RCTs targeting decisions related to diabetes reported significant changes in compliance with practice guidelines. In both studies, the CDSS was a part of integrated care interventions to improve evidence-based management of chronic conditions.  Reviewed evidence suggested that the effectiveness of CDSS is dependent on or can be predicted by the severity of patient presentation, type of clinical decisions, and type of decision support. It appears that CDSS were more effective in acute care than when less well-structured chronic care decisions were targeted by decision support. Considering the setting of care, all eight (100%) inpatient studies were positive, compared with only 5 of 16 (31%) primary care studies. These results may be confounded by the fact that many inpatient studies were concerned with prescribing which may be easier to optimise than some other clinical activities. At the same time, these findings probably reflect the higher impact of clinical decisions in acute care on patient outcomes.  More than half of the trials identified in our search showed a clinical benefit, and no study found the use of a CDSS to be detrimental. No RCTs exploring applications of the general medical diagnosis systems were identified in our search. Our findings confirmed previous observations that electronic decision support has the potential to improve the quality of clinical decisions, patients’ outcomes, and safety. However, the effectiveness of CDSS is not uniform. Controlled trials reviewed here strongly indicate that process measures, rather than “hard” patient outcomes, are more often accepted for evaluation of CDSS interventions. For example, the most common improvement in practice observed was the increase in adherence to clinical guidelines and protocols. The magnitude of the improvement in compliance ranged from 13% to 17% in a majority of successful interventions to a 227% increase in overall compliance with recommended diabetes care procedures in the prompted group of physicians in one study. | **Findings on moderators of effect**  Factors influencing success or failure of HIT systems were reviewed elsewhere. However, the role of decision tasks and different CDSS types on the impact of CDSS has received little attention. CDSS demonstrated modest effects in clinical trials with chronically ill patients where the relationship between clinical decision and outcome is less predictable. Critiquing CDSS have worked better, providing reminders for preventive care or assisting with drug prescribing. The usefulness of CDSS has likely arisen from their ability to use clinical information to answer straightforward questions, such as whether a certain drug was contraindicated or what was an appropriate dose for the medication. *Implementation of CDSS in these areas should be a high priority.* Consultative decision aids supporting the management of chronic illness in primary care showed a modest impact on the patient outcomes measured. None of the RCTs involving these CDSS demonstrated any significant improvement in Level 1 outcomes. It appears that clinical CDSS have a lesser effect on patients with chronic conditions than patient-level interventions such as computer-assisted insulin dose adjustment or utilisation of home glucose records for diabetes sufferers. Most of the successful trials in our review provided computerised guidelines to practitioners, or were designed as administrative aids for registration and recall. It is clear that the major factor limiting sustainable impact of CDSS on clinical practice is lack of knowledge of clinicians’ information processing, information needs and evidence. The absence of a significant effect on patient outcomes may reflect problems with the integration of systems within the clinical decision process or the level of CDSS adoption rather than unsatisfactory performance of a particular system itself. Only five trials reported on the adoption of CDSS, yet four of them documented low rates of the system usage. The majority of them studied electronic evidence-based guidelines for chronic conditions in a primary care setting where they may have been regarded as optional rather than standard. For example, computerised guidelines for diagnosis of hypertension and diabetes were used only in 12% of patients with diabetes in one controlled study, while 69% of patients did not receive the optimal therapy suggested by CDSS in another trial. It is plausible that the low uptake of CDSS in primary care studies significantly affected the negative findings of respective trials. It would be consistent with current evidence that CDSS use, and acceptance by healthcare practitioners remains low. | **Further research indicated**  Metrics more appropriate to CDSS evaluations  Evaluations using rigorous non-RCT designs, employing multi-disciplinary methods |

| **Reference & topic** Smith 2007 – Impact of CDSS for chronic pain management on practitioner performance and patient outcomes | | **Critical appraisal score**  17 |
| --- | --- | --- |
| **Studies and interventions**  9 non-experimental feasibility studies – 2000 to 2003  Both inpatient and outpatient settings    All systems were stand-alone, specific to a pain-related condition(s), using pain symptomology data | **Limitations of studies**  Validity: design, Hawthorne effect, power  Scope: inclusion of relevant processes/outcomes in evaluation  Utility: sufficient details on intervention and context provided | **Limitations of review**  Quantity: resource utilisation, practitioner performance, patient outcomes, organisational efficiency  Generalisability: inpatients  Validity: retrieval bias, inclusion/exclusion criteria  Evaluation of the clinical appropriateness of either the knowledge base, algorithms or output |
| **Findings on impact**  The majority of these studies, however, have focused exclusively on process measures, such as patient or clinician ratings of system acceptability and usability. Other salient process measures, such as the degree to which the clinician and/or patient actually reviewed and utilised system output, or had confidence in its accuracy, have not been consistently assessed. The effects of these systems on patient outcomes remain understudied. Other major outcomes, such as healthcare utilisation and costs, pain relief, pain medication usage, communication with healthcare provider about pain, functional status, and QOL, have not been examined. One study reported evidence that CDSS use may invoke patient reactivity (e.g. vomiting, intensified pain symptoms).  Due to insufficient data, definitive conclusions concerning the impact of decision support on practitioner performance and patient outcomes were not possible. | **Findings on moderators of effect**  Clinician perceptions concerning ease of use and value of a CDSS for chronic pain management were examined in two studies. Overall, physicians found the system to be moderately easy to use and of some clinical worth. | **Further research indicated**  Usage and usability  HFs, socio-technical issues on barriers/success  Powerful RCTs, practitioner performance, patient outcomes,  Adverse effects, primary care settings |

| **Reference & topic** Tan 2005 – Impact of CDSS on practitioner performance and patient outcomes in the neonatal care setting | | **Critical appraisal score**  19 |
| --- | --- | --- |
| **Studies and interventions**  3 RCTs – 1997 to 2001  2 on Rx and 1 on physiological monitoring | **Limitations of studies**  Validity: blinding, methodological reporting, metrics, power  Scope: inclusion of relevant processes/outcomes in evaluation  Utility: sufficient details on intervention and context provided | **Limitations of review**  Quantity: RCTs  Heterogeneity: intervention, intervention targets  Generalisability: older systems  Validity: retrieval bias, inclusion/exclusion criteria |
| **Findings on impact**  There are insufficient data from randomised trials to determine the benefits or harms of decision support in neonatal care. | **Findings on moderators of effect** | **Further research indicated**  RCTs |

| **Reference & topic** Thompson 2009 – Impact of EHRs in nursing on efficiency in the inpatient setting | | **Critical appraisal score**  9 |
| --- | --- | --- |
| **Studies and interventions**  5 MA and studies of unknown design – 2005 to 2009 | **Limitations of studies**  None noted | **Limitations of review**  None noted |
| **Findings on impact**  After reviewing most of the 11 articles in Poissant et al individually, we estimated that a 24% reduction in documentation time would equal 42 minutes per nurse per 12-hour shift in ICUs and 54 minutes in other nursing units. Three additional studies were found and after a more detailed review we concluded that, in some cases, patient-specific documentation time increases with electronic documentation; however, these increases may be offset by time-savings in other activities (e.g. care planning and coordination, order processing, shift change, MAR reconciliation) that become more efficient with electronic systems. It also appears that automated nursing documentation has ‘‘downstream’’ benefits to nursing efficiency that are not always evident in patient-specific documentation activities. We found only two articles (excluding two articles cited by Poissant et al) that reported no change in nursing efficiency after EHR implementation. We found no articles that reported an increase in overall nursing time requirements resulting from the use of information technology. Several other articles reported the results of nursing opinion surveys. In each of these nurses were reported as feeling that an EHR placed greater demands on their time, although they generally preferred automated documentation to paper. | **Findings on moderators of effect**  See figures 1 & 2 of original paper | **Further research indicated**  None noted |

| **Reference & topic** Uslu 2008 – Value of EHR in the inpatient setting | | **Critical appraisal score**  16 |
| --- | --- | --- |
| **Studies and interventions**  22 studies of variable design – 1991 to 2005  Majority in large US hospitals with academic affiliation | **Limitations of studies**  Validity: methodological reporting, power  Scope: inclusion of relevant processes/outcomes in evaluation | **Limitations of review**  Heterogeneity: settings, systems, hospital sizes  Validity: retrieval bias |
| **Findings on impact**  All the studies with the exception of show a direct economic benefit. The sole study that demonstrates a positive impact of the quality of care operates methodically at a medium level, the remaining three give at least positive indices, even if without numerical evidence.  For specific needs such as administration and data acquisition we identified good evidence for significant positive effects of EHRs. There is considerable evidence for a reduction of costs by the use of an EHR but little sign of an improvement in treatment quality. | **Findings on moderators of effect**  None noted | **Further research indicated**  Macro effects to determine value |

| **Reference & topic** van Rosse 2009 – Impact of eRx w/o on medication safety in the paediatric and intensive care setting | | **Critical appraisal score**  24 |
| --- | --- | --- |
| **Studies and interventions**  12 CTs, prospective and retrospective cohort studies – 2003 to 2007  Spread across adult ICU, N/P ICU and paediatric units, with the majority academic affiliates  Both commercial and home-grown systems | **Limitations of studies**  Scope: inclusion of relevant processes/outcomes in evaluation  Utility: sufficient details on intervention and context provided | **Limitations of review**  Quantity: paediatric ICU  Heterogeneity: systems, duration and timing of evaluation, metrics |
| **Findings on impact**  A meta-analysis PEs taking all studies together found a significant reduction in PEs (RR 0.08, 95%CI 0.01 to 0.77), uniformly observed in all studies, this reduction in PE did not directly result in reduction in clinically relevant ADEs (RR 0.65, 95%CI 0.40 to 1.08). However, there was significant heterogeneity (*I^2^* = 65%) among the studies. Meta-analysis of mortality rates were not significantly influenced by CPOE (RR 1.02, 95%CI 0.52 to 1.94). This was observed in all studies except for one with an RR of 2.35 (95%CI 1.51 to 3.65). Even after adjustment for possible confounders, the mortality risk remained elevated (OR 3.28, 95%CI 1.94 to 5.55). No new errors were demonstrated. | **Findings on moderators of effect**  Four studies described classroom training before implementation, extensive individualised instruction, and on-site support during and after CPOE implementation. Two of those studies showed a significant beneficial effect of CPOE. In the other two studies, authors reported three hours of classroom computer practice three months before implementation although positive effects on ADEs were shown so were negative effects on mortality. | **Further research indicated**  Standardised metrics and reporting of MEs with methods for their detection and evaluation clearly reported  CBAs across multiple sites  Immediately after implementation to assess learning curve |

| **Reference & topic** Wolfstadt 2008 – Impact of eRx/CDSS on medication safety | | **Critical appraisal score**  13 |
| --- | --- | --- |
| **Studies and interventions**  10 CTs, BAs and TSs – 1994 to 2007  Most in US inpatient settings  Majority from two benchmark institutions | **Limitations of studies**  None noted | **Limitations of review**  Quantity: RCTs, non-hospital settings    Generalisability: home-grown, older systems, limited sites  Heterogeneity: metrics |
| **Findings on impact**  eRx with decision support contributed to a statistically significant (p≤.05) decrease in ADEs in 5 of 10 studies. Four studies reported a non-statistically significant reduction in ADE rates, and one study demonstrated no change in ADE rates.  We know relatively little about the benefit of commercial systems on reducing ADEs; our results are similar to a previous systematic review. | **Findings on moderators of effect**  None noted | **Further research indicated**  Standardised metrics and reporting for ADEs  Cluster RCTs, commercials systems, long-term care settings, elderly, across multiple settings |

| **Reference & topic** Wong 2010 – Impact of eRx/CDSS on medication safety | | **Critical appraisal score**  22 |
| --- | --- | --- |
| **Studies and interventions**  4 RCTs, BAs and cohorts 1999 – 2005  Most in North American inpatient settings | **Limitations of studies**  Validity: randomization, blinding, uniformity of implementation | **Limitations of review**  Quantity: RCTs, non-hospital settings    Generalisability: home-grown, older systems, limited sites  Heterogeneity: metrics  Validity: retrieval bias |
| **Findings on impact**  The analysis showed a non-significant overall effect (RR 0.66, 95%CI 0.33 to 1.18). The posterior median I-squared was 52%, indicating insufficient information to assess the impact of study design on between-study heterogeneity.  Two sensitivity analyses were conducted, each removing one study from analysis (Tamblyn and Oliven). The results of the sensitivity analyses did not assist in reducing heterogeneity.  There is a distinct lack of evidence to support the touted benefits or cost-effectiveness of drug interaction software. | **Findings on moderators of effect**  None stated | **Further research indicated**  Interaction software |

| **Reference & topic** Yourman 2008 – Impact of eRx/CDSS in geriatrics on practitioner performance | | **Critical appraisal score**  16 |
| --- | --- | --- |
| **Studies and interventions**  10 RCTs, BA, ITS – 1998 to 2007  Majority in US outpatient settings  Majority provided direct support at the point of care and were not condition or disease-specific | **Limitations of studies**  None noted | **Limitations of review**  Heterogeneity: study designs and settings, systems, metrics  Validity: retrieval bias, publication bias |
| **Findings on impact**  Of those 10 studies evaluating eRx for older adults, 8 showed positive outcomes with regard to medication-related process measures. The NNT to improve prescribing ranged from 5 to 1960 among the studies with positive outcomes, with a median value of 33. The majority of studies reported medication-related process outcomes, for which CDSS generally showed positive effects such as lower rates of prescribing inappropriate drugs or higher adherence to better drug choices or dosages for older persons. Only a minority of CDSS studies reported any patient outcomes, and the effect on patient outcomes in older adults was much less clear. The studies reviewed here indicate that often straightforward point of care recommendations showed modestly effective results from a process outcomes perspective. | **Findings on moderators of effect**  Six of eight studies of direct support each found that prescribing outcomes in response to real-time recommendations were associated with improved medication use. | **Further research indicated**  On predictive value of process measures and ultimately more effective ways of measuring improvement in patient outcomes  Diverse settings to maximise potential to improve patient safety |
