# Supplementary material for: The Impact of eHealth on the Quality and Safety of Health Care: A Systematic Overview
Source: PLoS Med. 2011 Jan 18;8(1):e1000387. doi: 10.1371/journal.pmed.1000387 (PMC3022523; doi:10.1371/journal.pmed.1000387)
Supplement: Text S1 — Search strategy (databases, string, and filters). (0.05 MB DOC) [file pmed.1000387.s003.doc]

Text S1: Search strategy (databases, string and filters)

Databases searched**:** MEDLINE, EMBASE, Cochrane Library

Search string: Ovid (MEDLINE and EMBASE)

(Man-Machine Systems/ OR Office Automation/ OR Information Management/ OR Data Collection/ OR Automation/ OR Autoanalysis/ OR Technology, Radiologic/ OR Technology, Pharmaceutical/ OR Technology, Medical/ OR Point-of-Care Systems/ OR Patient Identification Systems/ OR Medication Systems, Hospital/ OR Hospital Communication Systems/ OR Adverse Drug Reaction Reporting Systems/ OR Biomedical Engineering/ OR Biomedical Technology/ OR Electronic Mail/ OR Emergency Medical Service Communication Systems/ OR Computing Methodologies/ OR pattern recognition, automated/ OR drug information services/ OR user-computer interface/ OR speech recognition software/ OR software/ OR numerical analysis, computer-assisted/ OR decision support techniques/ OR mathematical computing/ OR computer simulation/ OR Artificial intelligence/ OR Algorithms/ OR Feedback/ OR medical informatics/ OR Medical informatics applications/ OR Decision-making, computer-assisted/ OR diagnosis, computer-assisted/ OR image interpretation, computer-assisted/ OR radiographic image interpretation, computer-assisted/ OR therapy, computer-assisted/ OR drug therapy, computer-assisted/ OR "information storage and retrieval"/ OR information systems/ OR clinical laboratory information systems/ OR decision support systems, clinical/ OR hospital information systems/ OR medical order entry systems/ OR integrated advanced information management systems/ OR management information systems/ OR ambulatory care information systems/ OR clinical pharmacy information systems/ OR database management systems/ OR decision support systems, management/ OR operating room information systems/ OR "personnel staffing and scheduling information systems"/ OR radiology information systems/ OR medical records systems, computerized/ OR reminder systems/ OR medical informatics computing/ OR informatics/ OR Automatic Data Processing/ OR Public Health Informatics/ OR Nursing Informatics/ OR Patient Identification Systems/ OR Natural Language Processing/ OR Fuzzy Logic/ OR Expert Systems/ OR Knowledge Bases/ OR Medical History Taking/ OR "Neural Networks (Computer)"/ OR Programming, Linear/ OR Computers, Handheld/ OR "Appointments and Schedules"/ OR "Referral and Consultation"/ OR Information Services/

OR

(Clinical decision support OR Electronic outpatient booking OR Electronic refer$ OR electronic hospital refer$ OR Electronic discharg$ OR electronic patient discharg$ OR electronic health record$ OR computerized patient record$ OR computerised patient record$ OR personal health record$ OR Computerised intervention$ OR Computerized intervention$ OR IHCA OR Decision support technique$ OR Interactive Health Communications Application$ OR CDSS OR computer aid$ OR computer assisted OR Computer$ reminder$ OR Computerized Physician Order Entry OR CPOE OR data mining OR data repository OR e health OR eHealth OR eprescribing OR Electronic patient record OR electronic prescribing OR e-mail OR Electronic mail OR health informat$ OR health technology OR intranet OR PDA OR personal digital assistant OR information system$ OR Computerized laboratory results OR GP to GP OR GP 2 GP OR GP2GP OR GPtoGP OR Electronic laboratory results OR Clinical Laboratory information system OR Laboratory information system$ OR LIS OR Laboratory Information Management System OR Medical information systems OR Web based refer$ OR Internet-based refer$ OR e-Booking OR "Choose and book" OR Electronic prescri$ OR Electronic Transmission of Prescriptions OR Care Records Service OR "Picture Archiving and Communication System$" OR PACS OR QMAS OR "Quality Management and Analysis System$" OR bar cod$ OR (CAD and (computer-assisted OR computer-aid$))).tw.)

AND

(Diffusion of Innovation/ OR Efficiency, Organizational/ OR Models, Organizational/ OR "Organization and Administration"/ OR Organizational Culture/ OR Organizational Innovation/ OR Organizational Objectives/ OR Technology Transfer/ OR Attitude to Computers/ OR Computer Literacy/ OR Computer User Training/ OR Cost Savings/ OR Cost-Benefit Analysis/

OR

(usability OR sustain$ OR spread OR socio-technical OR sociotechnical OR implement$ OR evalaut$ OR computer anxiety OR change management OR change agent$ OR barrier$ OR agent of change OR adopt$).tw.

OR

(device approval/ OR equipment failure/ OR equipment failure analysis/ OR equipment safety/ OR exp health services misuse/ OR iatrogenic disease/ OR quality assurance, health-care/ OR quality control/ OR quality indicators, health-care/ OR quality of health-care/ OR risk reduction behavior/ OR software validation/ OR equipment design/ OR guideline adherence/ OR software design/ OR program evaluation/ OR total quality management/ OR (exp medical errors/ not exp observer variation/) OR (exp risk management/ not exp risk sharing, financial/) OR exp accident prevention/ OR exp "outcome and process assessment (health-care)"/ OR "Maintenance and Engineering, Hospital"/ OR "Forms and Records Control"/ OR "Facility regulation and control"/

OR

(wrong site surgery OR workaround OR underuse OR time out OR slip$ OR side effect$ OR sentinel event OR safety OR safe practice$ OR root cause OR red rule OR read back OR quality OR proximate cause OR production pressure OR product recall$ OR procedural deviation OR overriding alerts OR negligence OR near miss OR misuse OR mistake$ OR misdiagnosis OR medication reconciliation OR medical complication$ OR leapfrog OR adverse drug event$ OR adverse event$ OR adverse occurrence$ OR adverse reaction$ OR complication$ OR hazard$ OR failure$ OR incident$ OR improv$ OR error$ OR lapse OR information overload OR inappropriate OR human factors research OR human factors engineering OR human factors design OR human factors OR heuristic OR harm OR face validity OR Equipment failure OR delayed diagnosis OR defective product OR cost utility analysis OR cost benefit analysis OR contributing factor$ OR confirmation bias OR close call OR clinical governance OR availability bias OR appropriate treatment OR appropriate care OR alert fatigue OR adverse drug interaction OR iatrogenic OR swiss cheese model).tw.))

Search filters:

Scottish Intercollegiate Guidelines Network (SIGN) methodology filter for systematic reviews on Ovid MEDLINE

1. Meta-Analysis/
2. meta analy$.tw.
3. metaanaly$.tw.
4. meta analysis.pt.
5. (systematic adj (review$1 or overview$1)).tw.
6. exp Review Literature/
7. or/1-6
8. cochrane.ab.
9. embase.ab.
10. (psychlit or psyclit).ab.
11. (psychinfo or psycinfo).ab.
12. (cinahl or cinhal).ab.
13. science citation index.ab.
14. bids.ab.
15. cancerlit.ab.
16. or/8-15
17. reference list$.ab.
18. bibliograph$.ab.
19. hand-search$.ab.
20. relevant journals.ab.
21. manual search$.ab.
22. or/17-21
23. selection criteria.ab.
24. data extraction.ab.
25. 23 or 24
26. review.pt.
27. 25 and 26
28. comment.pt.
29. letter.pt.
30. editorial.pt.
31. animal/
32. human/
33. 31 not (31 and 32)
34. or/28-30,33
35. 7 or 16 or 22 or 27
36. 35 not 34

SIGN methodology filter for systematic reviews on Ovid EMBASE

1. exp Meta Analysis/
2. ((meta adj analy$) or metaanalys$).tw.
3. (systematic adj (review$1 or overview$1)).tw.
4. or/1-3
5. cancerlit.ab.
6. cochrane.ab.
7. embase.ab.
8. (psychlit or psyclit).ab.
9. (psychinfo or psycinfo).ab.
10. (cinahl or cinhal).ab.
11. science citation index.ab.
12. bids.ab.
13. or/5-12
14. reference lists.ab.
15. bibliograph$.ab.
16. hand-search$.ab.
17. manual search$.ab.
18. relevant journals.ab.
19. or/14-18
20. data extraction.ab.
21. selection criteria.ab.
22. 20 or 21
23. review.pt.
24. 22 and 23
25. letter.pt.
26. editorial.pt.
27. animal/
28. human/
29. 27 not (27 and 28)
30. or/25-26,29
31. 4 or 13 or 19 or 24
32. 31 not 30

SIGN methodology filter for randomised controlled trials on Ovid MEDLINE

1. Randomized controlled trials/
2. Randomized controlled trial.pt.
3. Random allocation/
4. Double blind method/
5. Single blind method/
6. Clinical trial.pt.
7. Exp clinical trials/
8. Or/1-7
9. (clinic$ adj trial$1).tw.
10. ((singl$ or doubl$ or treb$ or tripl$) adj (blind$3 or mask$3)).tw.
11. Placebos/
12. Placebo$.tw.
13. Randomly allocated.tw.
14. (allocated adj2 random).tw.
15. Or/9-14
16. 8 or 15
17. Case report.tw.
18. Letter.pt.
19. Historical article.pt.
20. Review of reported cases.pt.
21. Review, multicase.pt.
22. Or/17-21
23. 16 not 22

SIGN methodology filter for randomised controlled trials on Ovid EMBASE

1. Clinical trial/
2. Randomized controlled trial/
3. Randomization/
4. Single blind procedure/
5. Double blind procedure/
6. Crossover procedure/
7. Placebo/
8. Randomi?ed controlled trial$.tw.
9. Rct.tw.
10. Random allocation.tw.
11. Randomly allocated.tw.
12. Allocated randomly.tw.
13. (allocated adj2 random).tw.
14. Single blind$.tw.
15. Double blind$.tw.
16. ((treble or triple) adj (blind$).tw.
17. Placebo$.tw.
18. Prospective study/
19. Or/1-18
20. Case study/
21. Case report.tw.
22. Abstract report/ or letter/
23. Or/20-22
24. 19 not 23
