# Supplementary material for: The Impact of eHealth on the Quality and Safety of Health Care: A Systematic Overview
Source: PLoS Med. 2011 Jan 18;8(1):e1000387. doi: 10.1371/journal.pmed.1000387 (PMC3022523; doi:10.1371/journal.pmed.1000387)
Supplement: Text S2 — Intervention inclusion and exclusion criteria. (0.03 MB DOC) [file pmed.1000387.s004.doc]

**Text S2: Intervention inclusion and exclusion criteria**

Included interventions:

- use of computers in information exchange
- electronic health records
- computer-based history taking systems
- electronic booking systems and electronic referral systems
- computerised decision support systems
- artificial intelligence in healthcare (relevant to computerised decision support systems)
- computerised reminders in clinical practice
- computer-aided detection or diagnosis in medical imaging
- decision support for ePrescribing and other orders
- patient identification: bar-coding and biometric systems; biometric identification includes measuring and analysing human physical and behavioural characteristics for identification purposes (although we selected these studies their analysis has, with the agreement of the funders, not been undertaken because of time constraints; these important areas will we plan be reviewed in the context of a project extension)
- human factors related to computing in healthcare

Excluded interventions:

- computer-assisted and any other type of IT enhanced surgery
- computer-assisted therapy predominantly directed at or used by patients independently or under supervision of a healthcare professional (eg smoking cessation, cognitive behaviour therapy)
- telemedicine
- eLearning (the use of electronic technology and media to deliver, support and enhance learning and teaching)
- consumer health informatics (patient-oriented eHealth)
- information literacy
- point-of-care testing without CDSS or with CDSS directed at patients.
- public health surveillance systems
- eHealth systems implemented in developing countries
- references not having an a priori IT focus or objective but that reported on IT post retrieval of references
